# An Insight into the Transcriptome of the Digestive Tract of the Bloodsucking Bug, *Rhodnius prolixus*

**DOI:** 10.1371/journal.pntd.0002594

**Published:** 2014-01-09

**Authors:** José M. C. Ribeiro, Fernando A. Genta, Marcos H. F. Sorgine, Raquel Logullo, Rafael D. Mesquita, Gabriela O. Paiva-Silva, David Majerowicz, Marcelo Medeiros, Leonardo Koerich, Walter R. Terra, Clélia Ferreira, André C. Pimentel, Paulo M. Bisch, Daniel C. Leite, Michelle M. P. Diniz, João Lídio da S. G. V. Junior, Manuela L. Da Silva, Ricardo N. Araujo, Ana Caroline P. Gandara, Sébastien Brosson, Didier Salmon, Sabrina Bousbata, Natalia González-Caballero, Ariel Mariano Silber, Michele Alves-Bezerra, Katia C. Gondim, Mário Alberto C. Silva-Neto, Georgia C. Atella, Helena Araujo, Felipe A. Dias, Carla Polycarpo, Raquel J. Vionette-Amaral, Patrícia Fampa, Ana Claudia A. Melo, Aparecida S. Tanaka, Carsten Balczun, José Henrique M. Oliveira, Renata L. S. Gonçalves, Cristiano Lazoski, Rolando Rivera-Pomar, Luis Diambra, Günter A. Schaub, Elói S. Garcia, Patrícia Azambuja, Glória R. C. Braz, Pedro L. Oliveira

**Affiliations:** 1 Section of Vector Biology, Laboratory of Malaria and Vector Research, National Institute of Allergy and Infectious Diseases, National Institutes of Health, Rockville, Maryland, United States of America; 2 Instituto Nacional de Ciência e Tecnologia em Entomologia Molecular, Federal University of Rio de Janeiro, Rio de Janeiro, Brazil; 3 Instituto Oswaldo Cruz, Fundação Oswaldo Cruz, Rio de Janeiro, Rio de Janeiro, Brazil; 4 Instituto de Bioquímica Médica, Programa de Biotecnologia e Biologia Molecular, Universidade Federal do Rio de Janeiro, Rio de Janeiro, Brazil; 5 Department of Biochemistry, Institute of Chemistry, Federal University of Rio de Janeiro, Rio de Janeiro, Brazil; 6 Instituto Nacional de Metrologia Qualidade e Tecnologia, Diretoria de Metrologia Aplicada às Ciências da Vida, Programa de Biotecnologia, Prédio 27, CEP 25250-020, Duque de Caxias, Rio de Janeiro, Brazil; 7 Departamento de Genética, Instituto de Biologia, Universidade Federal do Rio de Janeiro, CEP 21944-970, Rio de Janeiro, Brazil; 8 Departamento de Bioquímica, Instituto de Química, Universidade de São Paulo, São Paulo, Brazil; 9 Instituto de Biofísica Carlos Chagas Filho, Universidade Federal do Rio de Janeiro, Rio de Janeiro, Brazil; 10 Center for Technological Innovation, Evandro Chagas Institute, Ananindeua, Pará, Brazil; 11 Departamento de Parasitologia do Instituto de Ciências Biológicas da Universidade Federal de Minas Gerais, Belo Horizonte, Minas Gerais, Brazil; 12 Institute for Molecular Biology and Medicine (IBMM), Université Libre de Bruxelles, Gosselies, Belgium; 13 Departamento de Parasitologia, Instituto de Ciências Biomédicas, Universidade de São Paulo, São Paulo, Brazil; 14 Institute for Biomedical Sciences, Federal University of Rio de Janeiro, Rio de Janeiro, Brazil; 15 Instituto de Biologia, DBA, UFRRJ, Seropédica, Rio de Janeiro, Brazil; 16 Departamento de Bioquímica, Escola Paulista de Medicina, Universidade Federal de São Paulo, São Paulo, Brazil; 17 Zoology/Parasitology Group, Ruhr-Universität, Bochum, Germany; 18 Centro Regional de Estudios Genomicos, Universidad Nacional de La Plata, Florencio Varela, Argentina; 19 Centro de Bioinvestigaciones, Universidad Nacional del Noroeste de Buenos Aires, Pergamino, Argentina; Yale School of Public Health, United States of America

## Abstract

The bloodsucking hemipteran *Rhodnius prolixus* is a vector of Chagas' disease, which affects 7–8 million people today in Latin America. In contrast to other hematophagous insects, the triatomine gut is compartmentalized into three segments that perform different functions during blood digestion. Here we report analysis of transcriptomes for each of the segments using pyrosequencing technology. Comparison of transcript frequency in digestive libraries with a whole-body library was used to evaluate expression levels. All classes of digestive enzymes were highly expressed, with a predominance of cysteine and aspartic proteinases, the latter showing a significant expansion through gene duplication. Although no protein digestion is known to occur in the anterior midgut (AM), protease transcripts were found, suggesting secretion as pro-enzymes, being possibly activated in the posterior midgut (PM). As expected, genes related to cytoskeleton, protein synthesis apparatus, protein traffic, and secretion were abundantly transcribed. Despite the absence of a chitinous peritrophic membrane in hemipterans - which have instead a lipidic perimicrovillar membrane lining over midgut epithelia - several gut-specific peritrophin transcripts were found, suggesting that these proteins perform functions other than being a structural component of the peritrophic membrane. Among immunity-related transcripts, while lysozymes and lectins were the most highly expressed, several genes belonging to the Toll pathway - found at low levels in the gut of most insects - were identified, contrasting with a low abundance of transcripts from IMD and STAT pathways. Analysis of transcripts related to lipid metabolism indicates that lipids play multiple roles, being a major energy source, a substrate for perimicrovillar membrane formation, and a source for hydrocarbons possibly to produce the wax layer of the hindgut. Transcripts related to amino acid metabolism showed an unanticipated priority for degradation of tyrosine, phenylalanine, and tryptophan. Analysis of transcripts related to signaling pathways suggested a role for MAP kinases, GTPases, and LKBP1/AMP kinases related to control of cell shape and polarity, possibly in connection with regulation of cell survival, response of pathogens and nutrients. Together, our findings present a new view of the triatomine digestive apparatus and will help us understand trypanosome interaction and allow insights into hemipteran metabolic adaptations to a blood-based diet.

## Author Summary

The bloodsucking bug *Rhodnius prolixus* is a vector of Chagas' disease, which affects 7–8 million people in Latin America. In contrast to other insects, the digestive tract of *Rhodnius* has three segments that perform different functions during blood digestion. Here we report analysis of transcriptomes for each of these segments using pyrosequencing technology amounting to several million sequences. Comparison of transcript frequency in digestive libraries with a whole-body library was used to evaluate expression levels, leading to the discovery of several families of enzymes associated with the digestion of proteins, carbohydrates, and lipids, as well as proteins involved in immunity, signal transduction, amino-acid metabolism, and detoxification. Together, our findings present a new view of the triatomine digestive apparatus and will help us understand the mechanism of blood digestion by *Rhodnius* and its interaction with the agent of Chagas' disease, *Trypanosoma cruzi*, a parasite that grows within the insect's digestive system.

## Introduction

Triatomine bugs belong to the family Reduviidae within the order Hemiptera (infra-order: Heteroptera), all instars of which feed exclusively on blood [1], [2]. Several species are vectors of Chagas' disease in the Americas, a chronic and debilitating disease, often fatal, which infects 7–8 million people in Latin America today [3]. Among the 140 triatomine species in five tribes [4], *Rhodnius prolixus—*a vector in Central and South America—became a model insect for insect physiology and biochemistry thanks to its use by Dr. Vincent Wigglesworth in the 1930s and onward [5]. Despite being a bloodfeeder, due to its taxonomic position, *R. prolixus* data are useful for researchers working with heteropteran agricultural pests [1]. Recently, its genome was targeted for sequencing, and included in this effort was the sequencing of several organ-specific cDNA libraries using pyrosequencing technology, which are described here.

The gut of triatomines differs from other hematophagous insects for which genomic data are available (mainly Diptera) because it is divided into three distinct segments (anterior midgut, AM; posterior midgut, PM and rectum, RE) that perform different functions during digestion of the blood meal and make this insect highly adapted for a blood meal. For example, a 30-mg *R. prolixus* V^th^ instar nymph can take 10 times its own weight in blood in fifteen minutes, the blood being stored in the bug's AM. Within seconds of initiating the meal, diuretic hormones and serotonin are released into the hemolymph triggering salt and water transport from the meal to the hemolymph, and into the Malpighian tubules and finally into the RE, thus concentrating the meal and reducing the bug's weight [5], [6]. Indeed, the bug's meal is reduced to its half by this urination within a few hours [5].

*R. prolixus* evolved from ancestors that on adapting to plant sap sucking lost their digestive serine proteinases and associated peritrophic membrane. This is a chitin-protein anatomical structure that may be synthesized by the whole or part of the midgut (type I) or by a ring of cells at the entrance of the midgut (type II). The peritrophic membrane envelops the food bolus in the midgut of most insects, leading to compartmentalization of the digestive process [7], [8]. Instead, the midgut cell microvilli in Hemiptera are ensheathed by a phospholipid membrane, the perimicrovillar membrane (PMM) [7], [9], which extends toward the midgut lumen with dead ends and, when collapsing, forms sheath packs [10]–[12]. PMMs were isolated from both *R. prolixus* [12] and *Dysdercus peruvianus* [13] midguts, leading to the identification of α-glucosidase as their biochemical enzyme marker. The presumed role of PMM was to absorb nutrients (mainly free amino acids) from the dilute sap ingested by the hemipteran and thysanopteran ancestors. On adapting to a diet rich in proteins, the heteropteran hemipteran (like *R. prolixus* and *D. peruvianus*) used lysosome-derived enzymes for digestion and the PMM as a substitute for the peritrophic membrane in the compartmentalization of digestion [7], [9], [12].

The AM additionally harbors an endosymbiont, *Rhodococcus rhodnii*, which is essential for the bugs' development and fertility [14]–[18]. The digestive tract is also where *Trypanosoma cruzi*, the protozoan agent of Chagas' disease, develops [19]. No proteolytic digestion occurs in the AM, where hemoglobin remains red in color for over a week after feeding, but where various endoglycosidases have been described [20]. Digestion of complex lipids, as triacylglycerol, is negligible in AM and takes place in the PM [21].

The AM slowly releases its contents into the PM over a period of ∼20 days, when the V^th^ instar nymph molts to an adult [5]. While most insects have trypsin-like enzymes, and an alkaline gut pH, for digesting proteins, Hemiptera have lysosomal-like cathepsins which are secreted into an acidic gut [22]. There are a negligible [23] and a major [24] cysteine proteinase that accounts for 85% of the total proteinase activity. This activity was initially interpreted as a cathepsin B but later was shown to include a cathepsin L-like proteinase [24], [25]. A cathepsin D-like proteinase accounts for the remaining midgut proteinase activity [24]. Amino and carboxypeptidases produce amino acids from the endopeptidase products [24], [26]. Toxic amounts of oxygen radical-producing heme are a byproduct of hemoglobin digestion, but these are stacked in the gut as a non-oxidizing form similar to the malarial pigment hemozoin. The stacking process in *R. prolixus* is dependent on the presence of PMM [27], [28].

The RE, like the mammalian bladder, possesses a transitional epithelium that can stretch to accommodate the feces and urine [5], [29]. It is from the rectal discharges that *T. cruzi* is released onto the mammalian host. The epithelia of the three gut segments are surrounded by smooth muscle [5].

As part of the *R. prolixus* genome sequencing effort several tissues in different post-feeding states and from different developmental stages were used to construct cDNA libraries which were submitted to pyrosequencing, including a whole body library (WB, 862,980 reads) and gut segment libraries from AM (156,780 reads), PM (145,986 reads) and RE (170,565 reads). Other tissues were also investigated, including fat body (FB, 177,944 reads), Malpighian tubule (MT, 186,149 reads), ovary (OV, 111,190), and testes (TE, 140,156 reads). These reads were assembled together into contigs, allowing identification of transcripts which are significantly overexpressed in particular tissues, thus allowing an insight on digestive organs' specific transcripts in *R. prolixus*. Additionally, over 2,900 coding sequences (CDS) were obtained, most (∼2,300) of them full length (Met to stop codon), which should help train the gene-finder programs for this organism and help characterize specifically transcribed genes in the *R. prolixus* digestive tract.

## Methods

### Ethics statement

All animal care and experimental protocols were conducted following the guidelines of the institutional care and use committee (Committee for Evaluation of Animal Use for Research from the Federal University of Rio de Janeiro, CAUAP-UFRJ) and the NIH Guide for the Care and Use of Laboratory Animals (ISBN 0-309-05377-3). The protocols were approved by CAUAP-UFRJ under registry #IBQM001. Technicians dedicated to the animal facility at the Institute of Medical Biochemistry (UFRJ) carried out all aspects related to rabbit husbandry under strict guidelines to insure careful and consistent handling of the animals.

### Insects

Insects used for transcriptome were *R. prolixus* from a colony kept at UFRJ (Rio de Janeiro), fed with rabbit blood, and raised at 28°C and 70% relative humidity. Adult females (five from each condition) receiving their second blood meal after the imaginal molt were dissected before feeding, twelve hours, twenty-four hours, two days, and five days after blood meal. A group of males (blood fed, five days after blood meal) was dissected to obtain testes. Organs (AM, PM, RE, FB, OV, MT, and TE) were dissected, homogenized in TriZol reagent (Invitrogen, San Diego, CA, USA), and processed as described below. To obtain a whole body (WB) library, nymphs and adults in several stages of feeding plus eggs were collected and extracted with TriZol, as follows: Eggs were collected at the day of oviposition and at days 2, 5 and 7 of development. First instars were collected at fasting (2 weeks after emergence) and at 2, 5 and 7 days after feeding (DAF); second and third instars were collected at fasting and at 2, 5, 7 and 9 DAF. Fourth instars were collected at fasting and at 2, 5, 7, 9 and 12 DAF. Fifth instars were collected at fasting and at 2, 5, 7, 9, 12, 14, 17 and 19 DAF. Adult males and females were collected at fasting and at 2, 5, 7, 9 and 12 DAF. All these 45 RNA preparations were pooled and used to obtain WB cDNA as described below.

### RNA extraction, library preparation, and sequencing

Organs were homogenized in TriZol reagent, and total RNA was isolated, followed by mRNA purification using the Micro-Fast track 2.0 kit from Invitrogen (San Diego, CA, USA) according to manufacturer's instructions. Libraries were constructed using the Smart cDNA Library Construction kit from Clontech (Palo Alto, CA, USA) and normalized using the Trimmer cDNA Normalization kit from Evrogen (Moscow, Russia).

The libraries were sequenced on a 454 genome sequencer FLX Titanium machine (Roche 454 Life Sciences, Branford, CT, USA).

### Bioinformatics

A detailed description of our bioinformatic pipeline can be found in our previous publication [30]. Pyrosequencing reads were extracted from vector and primer sequences by running VecScreen. The resulting assemblies plus the clean pyrosequenced data were joined by an iterative BLAST and cap3 assembler [30]. This assembler tracks all reads used for each contig, allowing deconvolution of the number of reads used from each library for tissue expression comparisons using a χ^2^ test. To compare gene expression between libraries, paired comparisons of their number of reads hitting each contig were calculated by X^2^ tests to detect significant differences between samples when the minimum expected value was larger than 5 and P<0.05. A 2-fold change (up or down) was considered of interest when statistically significant. Normalized fold ratios of the library reads were computed by adjusting the numerator by a factor based on the ratio of the total number of reads in each library, and adding one to the denominator to avoid division by zero. Notice that due to library normalization, the actually reported ratios are smaller than in reality. This assembled contigs can be browsed on Supporting Information S1 which is a hyperlinked excel file.

Coding sequences were extracted using an automated pipeline based on similarities to known proteins or by obtaining CDS from the larger open reading frame of the contigs containing a signal peptide. A non-redundant set of the coding and their protein sequences were mapped into a hyperlinked Excel spreadsheet, which is presented as Supporting Information S2. Signal peptide, transmembrane domains, furin cleavage sites, and mucin-type glycosylation were determined with software from the Center for Biological Sequence Analysis (Technical University of Denmark, Lyngby, Denmark). To assign coding sequences as being of bacterial, viral, or invertebrate origins, the top blastp scores of the deduced proteins against each database were compared. If the ratio between the top two scores was larger than 1.25 and the e value of the blastp against pathogen or vertebrate was smaller than 1e-15, then the CDS was assigned to the top-scoring organism group. This automatic analysis was followed up by manual verification.

Functional classification of the contigs and proteins was done using a program written by JMCR that takes in consideration a vocabulary of 280 words that are scanned against matches to the KOG, GO, CDD, SwissProt and NR databases, and assigned to 29 functional categories, as explained in [30]. The algorithm also takes in consideration the position of the word in the match description.

Sequence alignments were done with the ClustalX software package [31]. Phylogenetic analysis and statistical neighbor-joining bootstrap tests of the phylogenies were done with the Mega5 package [32].

Raw sequences were deposited on the Sequence Read Archive (SRA) from the NCBI under bioproject accession PRJNA191820. The individual run files received accession numbers SRR206936, SRR206937, SRR206938, SRR206946, SRR206947, SRR206948, SRR206952, SRR206983, and SRR206984. A total of 2,475 coding sequences and their translations were submitted to the Transcriptome Shotgun Assembly (TSA) project deposited at DDBJ/EMBL/GenBank under the accessions GAHY01000001-2475.

### Proteomic analysis

#### Solutions

All solvents and salts were of the highest quality available (HPLC Grade) from Biosolve LTD, SIGMA and Merck.

#### Sample preparation for SDS-PAGE

AM, PM and RE were dissected from five *Rhodnius* females 4 days after feeding on rabbit blood, washed two times in PBS (137 mM NaCl, 2.7 mM KCl, 17 mM NA_2_HPO_4_, 1.7 mM KH_2_PO_4_, pH 7.4) and lysed in 25 mM Tris-HCl (pH 7.5), 150 mM NaCl, 1% (w/v) CHAPS supplemented with protease inhibitors (Roche, Vilvoorde, Belgium) at 4°C for 1 h. The extract was centrifuged at 120,000 g at 4°C for 80 min. Proteins present in the resulting supernatant were called soluble proteins. The pellet was washed 3 times with 100 mM sodium carbonate buffer pH 11 to eliminate ribosomal proteins and then extracted two times with 25 mM Tris-HCl (pH 7,5), 150 mM NaCl, 1% (w/v) CHAPS, 1% (w/v) Triton X-114 supplemented with protease inhibitors at 4°C for 1 h. Triton-soluble proteins were called membrane proteins. Soluble and membrane proteins were precipitated with 100% ice-cold acetone overnight at −20°C. Pellets were centrifuged at 16,000 g for 15 min and washed two times with 80% ice-cold acetone. Proteins were separated on 4–12% (w/v) NuPAGE gels (Invitrogen, Merelbeke, Belgium) and revealed by SafeStain Coomassie Blue (Invitrogen, Merelbeke, Belgium).

#### Protein identification by LC-MS/MS

The protein bands from SDS-PAGE were excised, reduced, alkylated, and trypsin digested with sequencing grade modified trypsin (Promega, Leiden, Holland) as described previously [33]. The resulting peptides were fractionated by nano-flow LC using a 10 cm long×75 µm ID×3 µm C18 capillary column connected to an EASY-nLC (Proxeon Biosystems, Odense, Denmark) in tandem to a Waters mass spectrometer model QTOF Ultima Global (Waters, Zellik, Belgium). The elution was performed with a flow rate of 300 nl/min in a gradient of 10–50% solvent B in 35 min followed by 50–100% in 15 min (solvent A: 2% ACN/0.1% FA; solvent B: 98% ACN/0.1% FA) and directly analyzed on the Q-TOF. The full MS scan was collected in the positive ion mode in the mass range from 300–1200 m/z. The three most intense ions were submitted to CID with 15–40 V collision energy. Spectra were searched against *Rhodnius* annotated ORF sequences using in-house Mascot software (www.matrixscience.com). Database search parameters were the following: trypsin as the digestion enzyme (one miscleavage site allowed); 150 ppm for peptide mass tolerance; carbamidomethylation of cysteine residues and oxidation of methionine residues as fixed and variable modifications, respectively. Mascot individual search algorithms internal estimates using a 95% confidence cutoff was used. Protein identifications were then inspected manually for confirmation prior to acceptance. The mass spectrometry raw data have been deposited to PeptideAtlas public repository (http://www.peptideatlas.org/) with the identifier PASS00333.

#### Ion assignment to protein deduced from transcriptome

Results from Mascot search were exported as a CSV table to a DAT file containing the ions identified in each band. The peptides identified by MS were converted to Prosite block format [34] through a custom program. This data-containing file was used to search matches in the Fasta-formatted database of deduced proteins, using the Seedtop program, which is part of the BLAST package. The result of the Seedtop search was inserted into the hyperlinked spreadsheet (Supporting Information S3) to produce a hyperlinked text file with details of the match. This spreadsheet contains only the deduced proteins confirmed by at least two ions.

## Results and Discussion

### Library specifications and assembly

The 1,951,750 reads were assembled into 317,104 contigs and singletons, 66,010 of which had a length above 250 nt. These contigs are found in Supporting Information S1. Only this larger set was used in this work, which included a total of 1,641,334 reads, or 84% of the total. The assembly had 27,751 contigs larger than 499 nt, 8,324 contigs with lengths above 999 nt, and 972 above 1999 nt. Because the assembly algorithm included tracking of the reads, the number of reads resulting from each tissue could be accounted in the final contig, allowing for statistical tests of significant departure from expected values, namely χ^2^ tests. The nature of the RNA could be estimated by BLAST [35] comparisons to different databases, as indicated in the Methods section. We accordingly identified transcripts that were significantly more expressed in the whole digestive tract when compared to the WB library (Table 1), those more expressed in the AM when compared to the PM (Table 2), those more expressed in the PM when compared to the AM (Table 3), and those more expressed in the RE when compared to the combined AM+PM set (Table 4). Analysis was concentrated on contigs that were overexpressed in the digestive system with a P value<0.05; however, contigs related to selected specific aspects of midgut metabolism were also included in the analysis even when found at lower gut expression.

**Table 1:** Functional classification of gut-overexpressed transcripts (>10× compared to whole body) from *Rhodnius prolixus*.

Class	Number of contigs	Number of reads	Reads/contig	Percent reads
**Associated with digestive physiology**				
Digestive enzymes	25	12861	514.4	7.7
Transporters/storage	16	7532	470.8	4.5
Extracellular matrix/cell adhesion	12	4489	374.1	2.7
Mucins	8	8277	1034.6	5.0
Immunity	6	11306	1884.3	6.8
Lipocalins	6	4357	726.2	2.6
Other secreted	6	2175	362.5	1.3
Odorant binding proteins	4	557	139.3	0.3
Oxidant metabolism/detoxification	4	1683	420.8	1.0
Peritrophins	2	74	37.0	0.0
**Associated with cellular function**				
Cytoskeletal	13	21773	1674.8	13.1
Protein synthesis machinery	19	12286	646.6	7.4
Metabolism, energy	23	11184	486.3	6.7
Protein modification machinery	10	11092	1109.2	6.7
Proteasome machinery	15	9637	642.5	5.8
Unknown, conserved	44	5249	119.3	3.2
Nuclear regulation	4	2708	677.0	1.6
Transcription machinery	17	2260	132.9	1.4
Signal transduction	27	1902	70.4	1.1
Transcription factor	10	1858	185.8	1.1
Metabolism, intermediate	5	1387	277.4	0.8
Protein export machinery	11	1274	115.8	0.8
Metabolism, carbohydrate	5	541	108.2	0.3
Metabolism, lipid	6	462	77.0	0.3
Metabolism, amino acid	4	129	32.3	0.1
Metabolism, nucleotide	1	95	95.0	0.1
Nuclear export	1	17	17.0	0.0
Unknown	193	23028	119.3	13.8
Transposable element	12	6425	535.4	3.9
**Total**	509	166618		

**Table 2:** Functional classification of AM-overexpressed transcripts (>10× compared to posterior) from *Rhodnius prolixus*.

Class	Number of contigs	Number of reads	Reads/contig	Percent reads
**Associated with digestive physiology**				
Digestive enzymes	6	965	160.8	8.6
Protease inhibitors	1	266	266.0	2.4
Transporters/storage	4	223	55.8	2.0
Other secreted	1	104	104.0	0.9
Mucins	1	47	47.0	0.4
Oxidant metabolism/detoxification	1	32	32.0	0.3
**Associated with cellular function**				
Signal transduction	13	859	66.1	7.7
Transcription factor	3	722	240.7	6.5
Unknown, conserved	11	493	44.8	4.4
Cytoskeletal	3	466	155.3	4.2
Metabolism, amino acid	2	262	131.0	2.3
Protein export machinery	5	202	40.4	1.8
Transcription machinery	4	197	49.3	1.8
Metabolism, carbohydrate	2	90	45.0	0.8
Protein modification machinery	2	77	38.5	0.7
Metabolism, energy	1	56	56.0	0.5
Proteasome machinery	2	48	24.0	0.4
Unknown	68	5638	82.9	50.5
Transposable element	4	236	59.0	2.1
Viral	1	174	174.0	1.6
**Total**	135	11157		

**Table 3:** Functional classification of PM-overexpressed transcripts (>10× compared to AM) from *Rhodnius prolixus*.

Class	Number of contigs	Number of reads	Reads/contig	Percent reads
**Associated with digestive physiology**				
Other secreted	1	132	132.0	0.4
Transporters/storage	8	428	53.5	1.3
Digestive enzymes	22	8549	388.6	26.9
Mucins	2	3609	1804.5	11.4
Odorant binding proteins	4	1020	255.0	3.2
Immunity	2	325	162.5	1.0
Oxidant metabolism/detoxification	2	137	68.5	0.4
**Associated with cellular function**				
Nuclear regulation	2	148	74.0	0.5
Transcription factor	2	61	30.5	0.2
Transcription machinery	3	389	129.7	1.2
Protein synthesis machinery	5	129	25.8	0.4
Protein export machinery	2	72	36.0	0.2
Protein modification machinery	3	310	103.3	1.0
Proteasome machinery	1	64	64.0	0.2
Metabolism, carbohydrate	2	222	111.0	0.7
Metabolism, amino acid	2	45	22.5	0.1
Metabolism, lipid	2	266	133.0	0.8
Metabolism, intermediate	1	178	178.0	0.6
Signal transduction	7	191	27.3	0.6
Extracellular matrix/cell adhesion	6	4501	750.2	14.2
Cytoskeletal	5	187	37.4	0.6
Metabolism, energy	6	5158	859.7	16.2
Unknown, conserved	14	1109	79.2	3.5
Unknown	66	4527	68.6	14.2
Transposable element	1	29	29.0	0.1
**Total**	171	31786		

**Table 4:** Functional classification of RE-overexpressed transcripts (>10× compared to anterior + PMs) from *Rhodnius prolixus*.

Class	Number of contigs	Number of reads	Reads/contig	Percent reads
**Associated with digestive physiology**				
Transporters/storage	7	1292	184.6	3.6
Oxidant metabolism/detoxification	3	902	300.7	2.5
Other secreted	2	296	148.0	0.8
Digestive enzymes	3	244	81.3	0.7
Odorant binding proteins	3	193	64.3	0.5
Peritrophins	1	18	18.0	0.1
**Associated with cellular function**				
Cytoskeletal	17	4562	268.4	12.8
Transcription machinery	7	3803	543.3	10.7
Unknown, conserved	21	3351	159.6	9.4
Protein synthesis machinery	5	2130	426.0	6.0
Metabolism, amino acid	3	1656	552.0	4.7
Extracellular matrix/cell adhesion	8	1367	170.9	3.8
Metabolism, lipid	3	824	274.7	2.3
Metabolism, energy	6	704	117.3	2.0
Protein modification machinery	2	656	328.0	1.8
Signal transduction	6	554	92.3	1.6
Nuclear regulation	5	534	106.8	1.5
Transcription factor	1	462	462.0	1.3
Protein export machinery	5	457	91.4	1.3
Metabolism, carbohydrate	1	423	423.0	1.2
Proteasome machinery	2	214	107.0	0.6
Unknown	69	8609	124.8	24.2
Transposable element	4	2355	588.8	6.6
Total	184	35606		

We also made an effort to obtain coding sequences for all contigs that were significantly more expressed in the gut as well as for transcripts that presented >90% coverage with their best protein matches from the NR database, provided in Supporting Information S2, containing 2,570 CDS. The following sections highlight the gut-overexpressed transcripts but also include other CDS of related families for comparison. These are located in the several worksheets of Supporting Information S2 following the worksheet named RP-CDS. We will make frequent reference to the number of “reads” from the pyrosequencing runs, each read being one sequence unit that was used to assemble the contigs that are the subject of analysis. In the remainder of this paper, when mentioning a contig represented in Supporting Information S1, this will be indicated by Asb-### where ### is the contig number shown in column A. When reference is made to a CDS from Supporting Information S2, this will be indicated by RP-### where ### refers to the CDS number shown also in column A.

### Proteomic analysis

An exploratory proteomic analysis of *Rhodnius'* gut compartments was performed. The samples analyzed were prepared from insects fed on blood. The tissues were harvested on the fourth day after blood feeding. Regardless of this one point harvesting, about 10% of the proteins deduced from conceptual translation of the assembled 454 reads had their existence confirmed by this proteomic approach. Additional figure S1 shows the SDS-PAGE fractionation of membrane and soluble protein extracts obtained as described in methods from the tree compartments of *Rhodnius'* digestive tract. This figure exhibits the numbering of each fraction that was *in gel* digested and subsequently analyzed by mass spectrometry. The assignment of the ions produced by mass spectrometry to the deduced proteins was first done by the use of Mascot (www.matrixscience.com) and subsequently converted to Prosite block format as described in methods. This data-containing file was used to search matches in a formatted database of the deduced proteins, using the Seedtop program. The result of the Seedtop search was inserted into the hyperlinked spreadsheet (Supporting Information S3) to produce a hyperlinked text file with details of the match. Supporting Information S3 exhibits in columns CH to DE of the first worksheet the information that was considered as a confirmation of protein existence. The gel fraction number with larger coverage was assigned only when two or more ions were detected. The total number of fragments, including same ion when detected in more than one band, and the coverage in total amino acid residues without duplication is presented. To summarize these findings, Supporting Information S3 was created. This spreadsheet contains a subset of worksheet named CDS from Supporting Information S2 and is also hyperlinked to the information on the ions that corroborate the deduced proteins' existence. Additional table S1 is a table containing the functional classification of the deduced proteins confirmed through this proteomic approach. These proteins cover almost all classes that figures in tables 1–4. The rows in the spreadsheet presented as Supporting Information S3 were ordered alphabetically through column DG where this functional classification is presented. It is important to notice that eight proteins classified as unknown conserved were confirmed by this approach. This classification means that similar proteins have been found before in other species but no function has been assigned to them.

### Transcripts overexpressed in the digestive tract

The following sections are a guide to explore the several worksheets of Supporting Information S2 having the same names as the following headings:

#### Peritrophins

Peritrophins are structural proteins of the peritrophic membranes and are characterized by having one or more chitin-binding domains (CBDs) as defined by the consensus “CX_15–17_CX_5–6_CX_9_CX_12_ CX_6–7_C” [36]. Peritrophins may also contain highly glycosylated sections, named mucin domains [36]. The finding of typical peritrophins overexpressed in *R. prolixus* gut tissues is somewhat surprising, despite the fact that CBDs were found in proteins associated with cuticular structures such as trachea [37], hindgut and integument [38], [39]. CBD also occurs in some enzymes (like chitinase, chitin synthase, and chitin deacylase) which were removed from the list of peritrophins. Comparisons of transcript abundance between the AM vs. PM and the RE vs. AM+PM (Tables 2–4) show that each organ has its own set of overtranscribed peritrophins, indicating a tissue specialization of this protein family.

**Figure 1:**
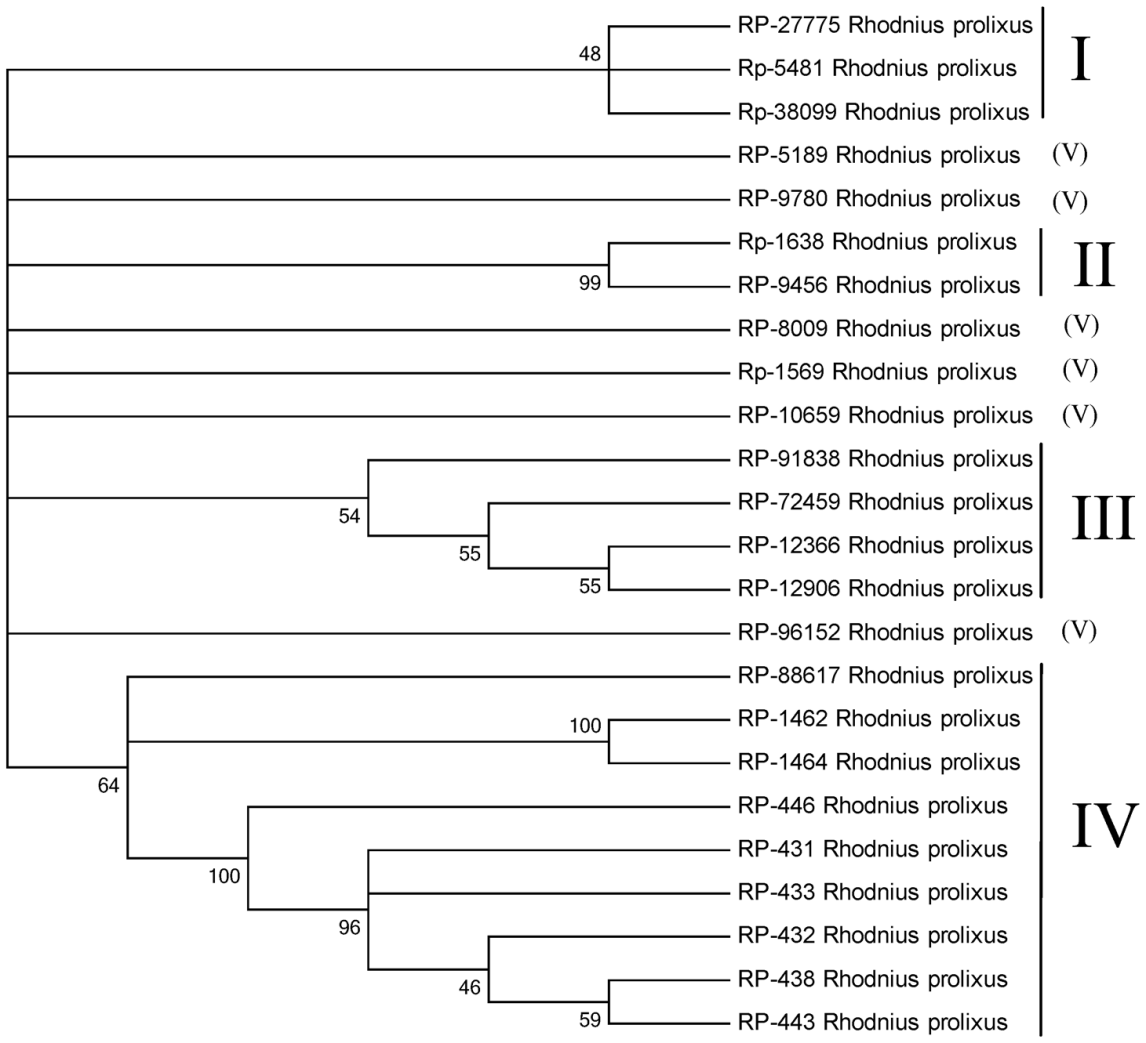
Cladogram of *Rhodnius prolixus* peritrophins. The dendrogram was generated with the neighbor-joining algorithm. Branches were statistically supported by bootstrap analysis (cut-off 45) based on ten thousand replicates. The Roman numerals indicate the perithrophin's group classification.

Peritrophins can be recognized by their signal peptide indicative of secretion and the domain pfam01607 (CBM_14), which corresponds to the CBD. Supporting Information S2 (spreadsheet) contains the coding sequence information for 38 proteins containing the CBM_14 domain, from which the most tissue differentially expressed proteins can be identified. Twenty four from the 38 sequences were complete and are further detailed here. Most of the sequences do not have mucin domains, as defined by Venancio et al. [40]; they may be divided into five groups (Fig. 1).

Group I (Fig. 1) contains peritrophins with 3 CBDs, although the third in the sequence has spaces between Cys residues similar to those of the cuticular proteins analogous to peritrophin 3 (CPA3) from *Tribolium castaneum* [39]. This - combined with the finding that they are overexpressed in WB and hindgut - favors the view they are a type of cuticular proteins.

Group II (Fig. 1) includes proteins with spaces between Cys residues distinct from the motif CX_15–17_CX_5–6_CX_9_CX_12_CX_6-–7_C. No motifs are retrieved from the conserved domain database (CDD) using rps-blast, although the software InterPro Scan (EMBL-EBI) found several CBDs.

Group III represents the proteins with one CBD that are highly expressed in tissues other than the midgut and, except for RP-72459, align with cuticular protein analogous to peritrophins 1 (CPA1) of *T. castaneum*.

Group IV is a set of nine proteins that includes three which are significantly overexpressed in the gut tissues, such as RP-431, with a total of 782 reads on the gut libraries and only 57 on the WB. This peritrophin is evenly expressed in the three gut libraries, being a good marker of gut tissue, as are RP-433 and RP-438. None of these is expressed in the FB, MT, or OV libraries, but they are expressed in the TE library. These proteins have a CBD that is preceded and followed by a sequence with several conserved Cys residues. This framework is also observed among the best-matching proteins found in the non-redundant (NR) protein database.

**Figure 2:**
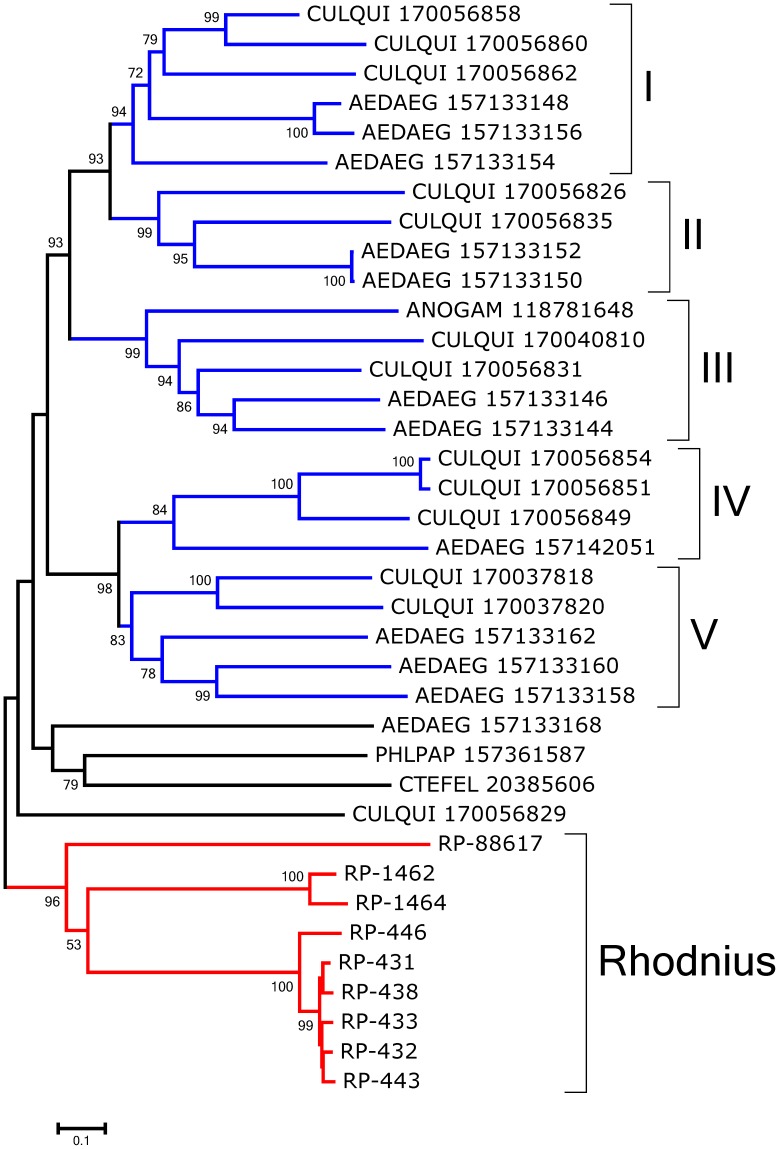
Bootstrapped phylogram of *Rhodnius prolixus* and other insect peritrophin annotated as Group IV peritrophin in Fig. 1. Bootstrap values above 50% are shown on the branches. The bottom line indicates 10% amino acid sequence divergence between the proteins. *R. prolixus* sequences are shown by the notation RP followed by a unique number. The remaining protein sequences were obtained from GenBank and are annotated with the first three letters of the genus name followed by the first three letters of the species name followed by their GenBank GI number. All non-*Rhodnius* sequences derive mostly from mosquitoes, with one deriving from a flea and another from a sand fly. Roman numerals indicate clades with mixed mosquito genera. Ten thousand replicates were done for the bootstrap test using the neighbor joining method.

The bootstrapped phylogram of Group IV peritrofins aligned with closely related sequences from other insects (Fig. 2) shows all *R. prolixus* sequences fall within a single clade with strong bootstrap support, supporting the existence of at least three genes that differ more than 50% in sequence identity. The sequences RP-431, RP-434, RP-433, and RP-438 may be alleles. Notice also that the mosquitoes *Aedes aegypti* and *Culex quinquefasciatus* - shown in Fig. 2 - have indications of at least five different genes with families that diverged before the separation of their genera as indicated by clades containing both genera and having strong bootstrap support (marked I–V in Fig. 2). Quite interestingly, all the proteins collected in this group are from bloodsucking insects that do not share a common bloodsucking ancestor with *Rhodnius*, suggesting either convergent evolution or gene expansion of a common insect gene when associated with blood feeding. All the proteins of this group are predicted to be secreted except RP-88617 and RP-1462, which are predicted to lack a signal peptide or to be membrane-bound, respectively. Once in the midgut lumen, these proteins may bind heme, as AeIMUCI [41], possibly to catalyze the formation of hemozoin.

Group V corresponds to proteins that do not form a monophyletic clade in Fig. 1. They are probably cuticular proteins, as discussed for sequences from Groups I and III.

Supporting Information S2 (worksheet “Peritrophins”) lists other proteins of this class, not necessarily with significant tissue differential expression.

#### Vertebrate-like mucins and other secreted proteins

The term mucin denotes two different molecules. Mucin may correspond to a highly glycosylated Ser+Thr-rich protein such as vertebrate mucin [42] or name a peritrophin with a very long mucin domain [43]. *R. prolixus* mucins referred to here correspond to the first type. Thus, RP-5412 codes for a Ser+Thr-rich protein with 70 putative N-acetyl-galactosamination sites. Its low complexity makes it difficult to assess close eukaryotic proteins, the best match by blastp to the NR database (with the filter of low complexity off) being with a bacterial protein. It is represented by 141 digestive transcripts and only 27 WB reads. RP-3746 and RP-3448 are overtranscribed somewhat equally in the three digestive tissues, while RP-15656 is overexpressed in the AM, where 43 of the 45 reads from the digestive tissues derive, none being found in the WB, but two from the TE. The worksheet “Mucins” in Supporting Information S2 contains these and a few other mucins.

The Smart ML domain predicts proteins involved with innate immunity and lipid metabolism. It is similar to the KOG domain for the major epididymal secretory protein HE1 and the PFAM E1_DerP2_DerF2 domain implicated in recognition of pathogen-related products. RP-5669 has such a domain and is 11.5-fold overexpressed in gut tissues. Five other transcripts are shown on the worksheet “Other” of Supporting Information S2, including homologs of accessory gland proteins and other proteins found in *Triatoma* sialotranscriptomes and in the midgut transcriptome of sand flies, with unknown function.

#### Digestive enzymes

Carbohydrate digestion: It has been previously proposed that the digestive glycosidases of *R. prolixus* could help in digesting their endosymbiont cell walls [20]. Glycosidases could also have some importance in vector-parasite interactions, as several parasite surface molecules are heavily glycosylated. Glycosidases are classified in glycoside hydrolase families (GHFs) according to their amino acid sequence similarities (Carbohydrate Active Enzymes database, at http://www.cazy.org/; [44]). The worksheet “Carb digest” in Supporting Information S2 shows several of these enzymes, four of which are >10-fold overexpressed in digestive tissues. They comprise 13 enzymes belonging to nine different GHFs, namely families 1, 9, 13, 20, 29, 31, 35, 38, and 63.

**Figure 3:**
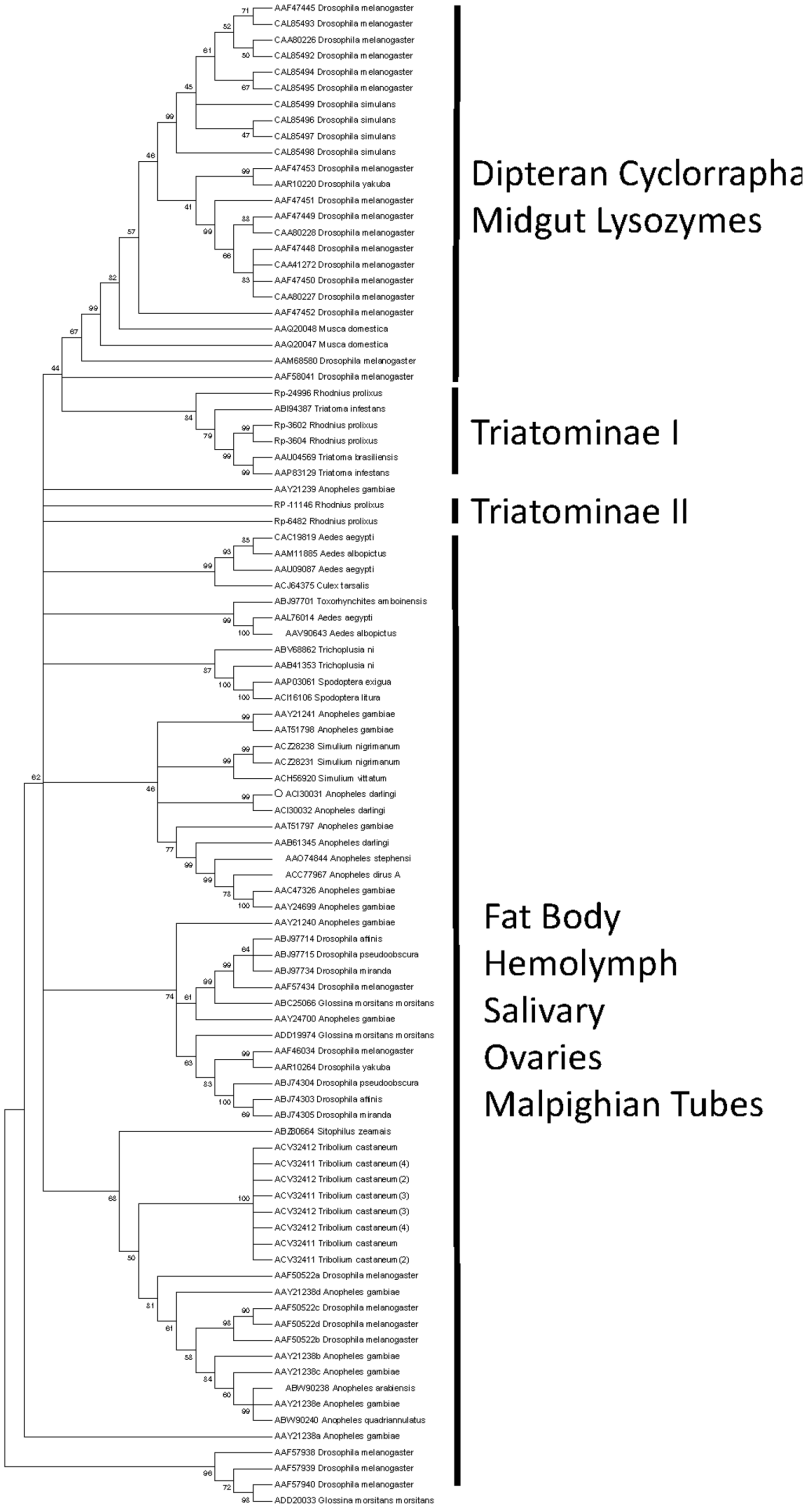
Cladogram of insect Lysozymes from glycoside hydrolase Family 22. The *R. prolixus* sequences are shown by the notation RP- followed by a unique number. The remaining proteins were obtained from GenBank and they are annotated with accession number followed by species name. The dendrogram was generated with the UPGMA algorithm. The branches were statistically supported by bootstrap analysis (cut-off 40) based on 1,000 replicates.

The two hexosaminidases highly expressed in the *R. prolixus* midgut (RP-29656 and RP-25051) belong to family 20 of glycosyl hydrolases. Insect hexosaminidases from family 20 were already described as secreted or cytosolic enzymes [45], but in the case of *R. prolixus* enzymes, this information could not be assessed due to the lack of 5′ sequence in both contigs. Interestingly, insect hexosaminidases are related to mammalian lysosomal hexosaminidases, which raises the possibility that they were originally lysosomal enzymes recruited for digestion during the evolution of Hemiptera, as has been suggested already for proteolytic enzymes [7]. RP-25051 shares the catalytic residues Asp240 His294 Glu355 with human hexosaminidase but this information is lacking for RP-29656. These proteins can be involved in the digestion of N-linked oligosaccharides. RP-25051, however, does not seem to be exclusively digestive (141 reads in WB and 33 in gut libraries, 25 from RE). In contrast, RP-29656 has 19 reads, all from gut libraries, especially from AM. The distinct patterns of expression displayed by these two transcripts indicate distinct roles for these two proteins. These roles could correspond to the initial digestion of glycoproteins and intermediate or final digestion of chitin or bacterial cell wall polysaccharides, which would be consistent with the distinct compartmentalization of these two GHF20 proteins. In this respect, the expression of β-hexosaminidases should be concomitant with the production of chitinase, lysozymes, and proteinases. No chitinase is included in the set of highly transcribed midgut genes. In fact, from the four chitinases present in the whole-body screening (all from GHF18), only one showed significant expression in the gut (RP-13146), but this transcript belongs to insect chitinase family V, which is related to Imaginal Growth Factors (IGFs) and has no described catalytic role [46]. It is unlikely that this *R. prolixus* IGF has catalytic activity, because its sequence lacks the glutamate identified as the catalytic proton donor in other family 18 chitinases, which in this case is substituted by a glutamine residue. Nevertheless, a highly active chitinase was recently purified and characterized from *R. prolixus* midgut (Genta, F.A., not published), but this activity seems to be secreted at later stages of blood digestion, which were not screened in this study. Perhaps the high lysozyme activity observed at later stages of digestion can account for the observed chitinase activity [20], since lysozyme have substantial chitinase activity in addition to hydrolyzing peptidoglycan [47]. It seems more likely that *R. prolixus* hexosaminidases act on lysozyme products, as five of these proteins, belonging to GHF22, are highly expressed in the gut (RP-3602, RP-3604, RP-6482, RP-11146, and RP-24996, further discussed in the section on immune-related transcripts). Phylogenetic analysis of insect proteins from GHF22 (Fig. 3) reveals that only three *R. prolixus* GHF22 sequences (RP-24966, RP-3602, and RP-3604) group with other triatomine gut proteins (Triatomine clade I). In spite of that, they do not group with the other described insect digestive lysozymes from Diptera: Cyclorrapha, mainly from *Musca domestica* [48] and *Drosophila melanogaster* [49]. This suggests that some adaptive convergence could have occurred in these two insect groups, with the recruitment of lysozymes for digestion of bacteria. In the case of *R. prolixus*, digestion of the symbiont *R. rhodnii* seems to be a probable function of these enzymes.

The finding of a glycoside hydrolase from family 9 in *R. prolixus* (RP-10367; 4 reads from WB and 74 reads in gut, exclusively in PM) is quite unexpected, as GHF9 that were described in termites, beetles, and cockroaches are mainly cellulases (endo-β-1,4-glucanases) involved in plant cell-wall digestion [50]; however, GHF9 also contains several β-glycosidases, and it is difficult to ascertain a specificity or action pattern for these enzymes based only on a partial sequence. Two α-mannosidases transcripts were identified: RP-3116 is markedly digestive with 65 reads in the gut, coming from PM and RE, and only 4 reads in WB and RP-2863, which showed 46 reads from WB and 37 reads coming from all three gut libraries. They belong to GHFs 38 and 63, respectively. Family 38 contains only mannosidases, mainly from lysosomal origin, which reinforces the use of lysosomal glycosidases in *R. prolixus* digestion. Family 63, a poorly described glycoside family in eukaryotes, contains several α-glucosidases as well, making it difficult to construe the specificity or function to this member.

A complete sequence of a typical α-amylase (RP-10100) was found that is expressed mainly in AM. This amylase is predicted to be activated by chloride ions and because of this, it should not be responsible for the amylase previously assayed in *R. prolixus* AM, which is secreted by *R. rhodnii* and is not activated by these ions [24]. From the four amylases highly expressed in the midgut (RP-10100, RP-8390, RP-5922, and RP-3792), three are from family 13 and only one (RP-5922) from family 31, which is related to α-glucosidases. RP-3792 has the same conserved catalytic residues of α-amylase but does not show complete calcium and chloride pockets, suggesting it is an α-glucosidase. As this sequence has a predicted signal peptide and GPI-anchor, it is a good candidate to correspond to the α-glucosidase activity that is a marker enzyme of the perimicrovillar membranes [51]. RP-10100 is a full-length transcript coding for an α-amylase overexpressed in gut tissues, mainly in AM (53 reads against only 9 reads from WB). While RP-10100 is more expressed in AM, RP-8390 and RP-3792 are more expressed in the PM. This could be related to different phases of polysaccharide digestion, corresponding to differences in the action pattern of these enzymes, e.g., liquefying or saccharifying amylases. As *R. prolixus* is strictly hematophagous, the nature of the physiologic substrate of these enzymes remains unclear. An α-glucosidase from family 13 has been implicated in formation of hemozoin in the *Rhodnius* midgut [52], but no transcript coding for that enzyme (accession # FJ236283) was found here. The presence of several enzymes of this group raises the possibility that more than one protein may act in seeding formation of hemozoin crystals.

*R. prolixus* midgut β-glycosidases are members of GHFs 1 (RP-12000 and RP-16121) and 35 (RP-4801). Family 35 members are mainly β-galactosidases, and family 1 contains enzymes with different β-glycosidase specificities. RP-12000 has a signal peptide and a GPI anchor and therefore can account for the β-glucosidase activity associated with the midgut cell microvillar membrane Insect ß-glycosidases can be divided into two classes. Class A includes the enzymes that hydrolyse substrates with hydrophilic aglycones, as disaccharides and oligosaccharides. Class B comprises enzymes that have high activity only on substrates with hydrophobic aglycones, such as alkyl-, p-nitrophenyl-, and methylumbelliferyl-glycosides [47]. The physiological role of these β-glycosidases is thought to be the digestion of oligosaccharides and glycolipids, respectively [53]. It is possible that *R. prolixus* has three active midgut β-glycosidases (two β-glucosidases and one β-galactosidase) fulfilling these two roles, a situation already described in several insects [53].

One transcript coding for an α-fucosidase (RP-6619) pertains to GHF 29 and probably is involved in the release of L-fucose residues from oligosaccharide moieties attached to glycoproteins. The coding sequences for these and other carbohydrate-hydrolyzing enzymes are shown on the worksheet “Carb digest” within Supporting Information S2.

Polypeptide digestion: Aspartyl and cysteinyl protease-coding transcripts dominate among those that are significantly overtranscribed in the gut tissues. Interestingly, despite no blood digestion being detected on the AM [54], several of those proteinases are highly expressed in the AM as well as in the RE, in addition of the PM. For example, the aspartyl protease coded by RP-2217 hits 2,857 reads from the digestive tract, and only 72 from the WB. From these 2,857 reads, 1,113 are from the AM, while 609 and 1,135 are from the PM and RE, respectively. A similar profile occurs with RP-2814. Also two different aspartyl proteases-encoding transcripts of *Triatoma infestans*—*TiCatD* and *TiCatD2*—were both expressed in AM and PM but active proteases were only isolated from PM [2] . Expression of aspartyl proteases in the AM can be interpreted as expression of pro-enzymes, such as pepsinogen, that might be activated in the PM. Alternatively, at least part of these enzymes, as well those expressed in RE (which epithelial cells are covered with a cuticle), may play intracellular roles.

**Figure 4:**
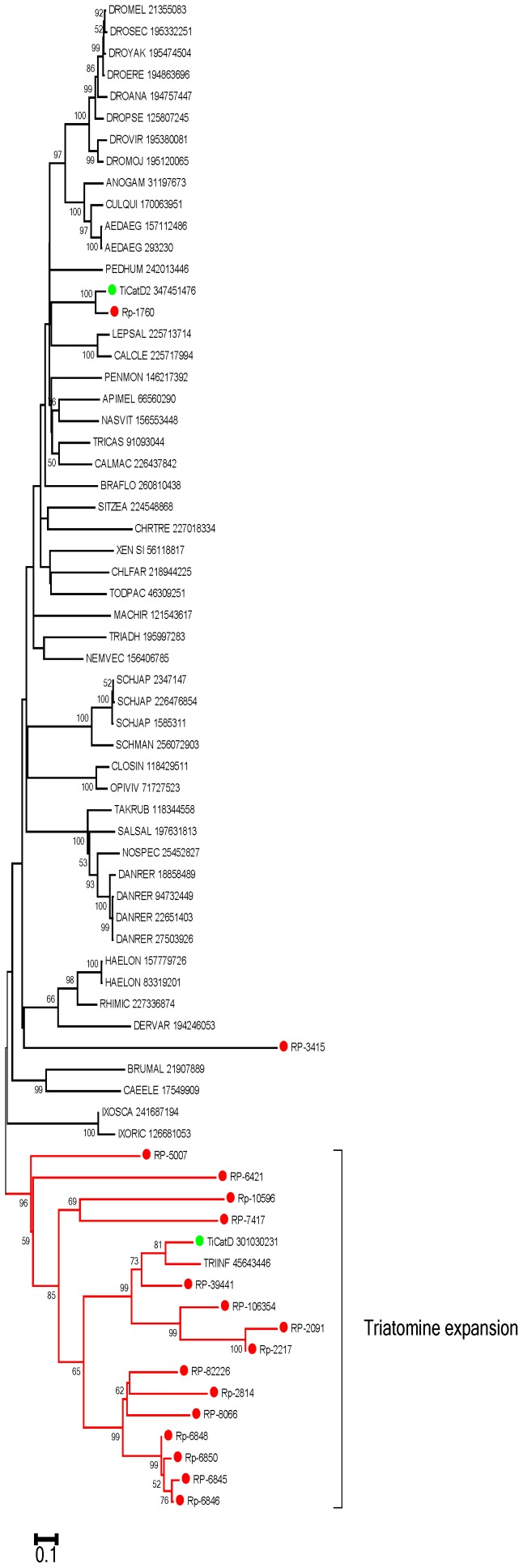
Bootstrapped phylogram of *Rhodnius prolixus* and other aspartyl proteinases. Bootstrap values above 50% are shown on the branches. The bottom line indicates 10% amino acid sequence divergence between the proteins. *R. prolixus* sequences are shown by the notation RP followed by a unique number and have a red circle preceding their names. The *Triatoma infestans* sequences from Balczun et. al. [2] have a green marker. The remaining sequences were obtained from GenBank and are annotated with the first three letters of the genus name, followed by the first three letters of the species name, followed by their GenBank GI number. One thousand replicates were done for the bootstrap test using the neighbor joining test.

The worksheet “Proteases” of Supporting Information S2 provides for 17 coding sequences from aspartyl proteases, most of them full length. All the aspartyl proteinases listed are actually cathepsin D-like enzymes. The motif [DxPxPx(G/A)P] - the proline loop - was suggested to be characteristic for lysosomal cathepsin D-like enzymes which were not secreted into the lumen of the digestive tract, because this motif is absent in digestive enzymes such as pepsin in vertebrates and digestive cathepsin D in cyclorrhaphan flies [55]. However, according to mass spectrometry of proteins from the lumen of the PM of *T. infestans* and the sequencing of the respective genes, one cathepsin D without (TiCatD) and one with the entire proline loop (TiCatD2) are present in the lumen [2]. In contrast to the expression of *TiCatD*, that of *TiCatD2* changes only slightly after feeding, indicating different roles of both enzymes [2]. TiCatD is putatively a digestive enzyme, whereas the role of TiCatD2 remains unclear, although it branches with lysosomal enzymes in Fig. 4. RP-1760 is the only *R. prolixus* sequence that has a proline loop and, although it may be a conserved lysosomal enzyme based on this evidence, also supported by its branching pattern in Fig. 4, it may be partially found in lumen as TiCatD2 [12]. It is worth mentioning that enzymes like lysosomal acid phosphatase are partially discharged into midgut lumen [12]. RP-3415 and RP-2091 are probably non-digestive cathepsin Ds, the first because it misses most of the conserved residues that form the subsite binding pockets, and the second because it lacks the first catalytic residue in the sequence. RP-5007 has an incomplete (DxP) proline loop, which suggests a special function unknown until now. All the other sequences lack the proline loop and are, thus, candidates to be responsible for the midgut cathepsin D activity in *R. prolixus*.

Analysis of the *R. prolixus* aspartyl proteases aligned with their best-matching proteins from GenBank produces a phylogram (Fig. 4) showing most (13) of the *R. prolixus* sequences forming a single clade, which includes a *Triatoma infestans* sequence. This *T. infestans* sequence - like those of *R. prolixus -* lacks the proline loop. This triatomine gene expansion is indicative of divergence and gene conversion, suggesting this cluster of proteins originates from a chromosomal tandem array. This phenomenon probably occurred in the heteropteran ancestors. The aspartyl proteases RP-1760 and TiCatD2 exceptionally group with other vertebrate and invertebrate proteins, arguably lysosomal enzymes, despite RP-1760 being overexpressed in the *R. prolixus* midgut.

Transcripts coding for three cysteinyl proteases are overexpressed in the digestive tissues, RP-1305 being assembled from 97 transcripts from the WB and 761 from digestive tissues, 707 of which derive from the PM, allowing for the identification of its entire CDS. RP-2313 and RP-1304 are also overexpressed in the digestive tissues—especially in PM. Regarding these three cysteinyl proteases abundantly expressed in gut tissues, only 1 read is found for the TE library, suggesting that the reads from this organ that have a digestive expression (peritrophins, mucins, and aspartyl proteases) do not derive from tissue contamination. Several other transcripts coding for cysteinyl proteases are found with larger expression in the PM when compared to the AM, despite being also found in the WB. The worksheet “Proteases” (Supporting Information S2) presents the CDS of 11 cysteinyl proteases, mostly full length.

**Figure 5:**
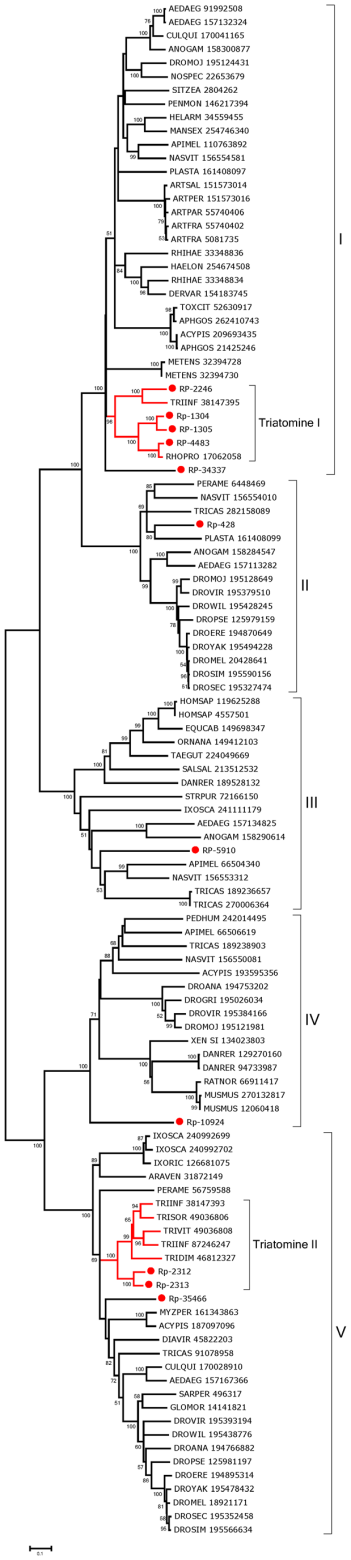
Bootstrapped phylogram of *Rhodnius prolixus* and other cysteinyl proteinases. Bootstrap values above 50% are shown on the branches. The bottom line indicates 10% amino acid sequence divergence between the proteins. *R. prolixus* sequences are shown by the notation RP followed by a unique number and have a red circle preceding their names. The remaining sequences, obtained from GenBank, are annotated with the first three letters of the genus name, followed by the first three letters of the species name, followed by their GenBank GI number. One thousand replicates were done for the bootstrap test using the neighbor joining test.

All of these cysteinyl proteases possess the presumed active triad residues that are characteristic of this class of proteases, namely cysteine, histidine, and asparagine, except for RP-10924, which lacks the cysteine residue and is therefore of unknown function. In addition, the glutamine residue attributed to the oxyanion binding site is present in all proteases. Phylogenetic analysis of these cysteinyl proteases indicates two triatomine gene subclades, noted as Triatomine I and II within clades I and V (Fig. 5), with an addition of three proteins scattered in other clades. Within the Triatomine I subclade, the protein with accession number gi|17062058 was previously reported as expressed in the guts of I- to IV-stage nymphs but not in the V^th^ stage, and as typical of a cathepsin L-type of cysteinyl protease [56]. Also in this subclade is found a *T. infestans* protein (gi|38147395), reported previously as a digestive cathepsin L [25]. The triatomine II subclade contains several enzymes previously reported from the genus *Triatoma* as having similarity to cathepsin B, such as gi|38147393 and gi|87246247 from *T. infestans* [25], and from other triatomines, as listed in Fig. 5. These enzymes possess the occluding loop, a structure characterizing them as cathepsin B proteases and being responsible for switching from endopeptidase to exopeptidase activity [57]. The sequence RP-428 within clade II, although overexpressed in the gut tissues, is only mildly so at 2.4 times the expected neutral value and may be an enzyme working in lysosomal rather than a secreted digestive function. Similarly, RP-5910, within clade III is not overexpressed in gut tissues. RP-34337—which belongs to clade I but not to the triatomine I subclade—is actually overexpressed in the WB library as compared with the digestive tract, which had only 1 read as opposed to 40 reads from the WB.

A CDS expressing a cathepsin F is presented in the form of RP-1287, overexpressed (12 fold) in gut tissues. Interestingly, this protein has four cystatin domains in its amino terminus followed by a typical papain-like domain, a structure that is conserved in human proteins as well [58], [59], indicating it is an ancient gene structure.

**Figure 6:**
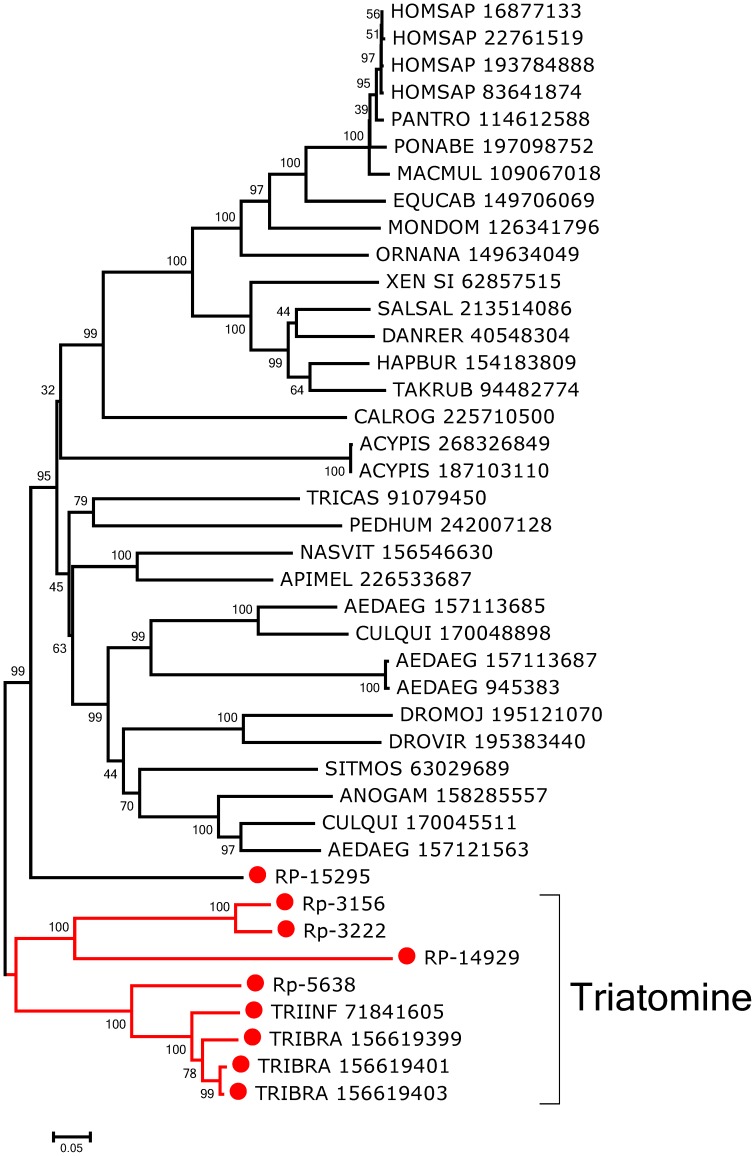
Bootstrapped phylogram of *Rhodnius prolixus* and other carboxypeptidases. Bootstrap values above 50% are shown on the branches. The bottom line indicates 10% amino acid sequence divergence between the proteins. *R. prolixus* sequences are shown by the notation RP followed by a unique number and have a red circle preceding their names. The remaining sequences were obtained from GenBank and are annotated with the first three letters of the genus name, followed by the first three letters of the species name, followed by their GenBank GI number. One thousand replicates were done for the bootstrap test using the neighbor joining test.

Two CDS represent the carboxy region of trypsin-like serine proteases. RP-2259 showed only 64 reads from WB and 2,851 hits from gut tissues, 2,346 of these being from the RE, 504 from the PM, and only 1 read from the AM. RP-19173 is also overexpressed in the RE, where 154 of the 181 gut-derived reads originate. RP-19173 is also well expressed in the WB, with 141 reads. Trypsin-like serine proteases were found in the salivary glands of *T. infestans* and *Panstrongylus megistus* [60], [61] but no trypsin activity has been reported in the digestive tract of triatomine insects. These data—together with the predominance of cysteine and aspartic proteinases and the marked overexpression in RE—indicates that these enzymes will not have a digestive role, but act in the cells of the intestinal wall. Five carboxypeptidases containing the PFAM peptidase S10 domain are shown in the “Proteases” worksheet of Supporting Information S2, including RP-5638, which is overexpressed in the PM, and RP-3222, overexpressed in the AM. All these enzymes contain the catalytic triad of residues of a serine, aspartate, and histidine. Phylogenetic analysis of these carboxypeptidases aligned with their matches to the GenBank proteins shows distinct triatomine clades that do not group with any other sequences with significant bootstrap support except for RP-15295, which groups with 99% support in an animal clade (Fig. 6). The other triatomine sequences derive from *T. infestans* and from *Triatoma brasiliensis*. RP-15295, outside this triatomine clade, is underexpressed in the digestive tissues when compared to the WB, and may not have a specific digestive function.

Two additional terminal peptidases are conspicuously absent from the AM but present in PM and RE (RP-5555 and RP-2304, both full length). Both present the PFAM peptidase_S28 domain contained in the enzymes lysosomal Pro-X carboxypeptidase, dipeptidyl-peptidase II, and thymus-specific serine peptidase.

Three other peptidases that are significantly overexpressed in the digestive tract as compared to the WB, or between digestive organs are noted in the worksheet “Proteases” of Supporting Information S2.

#### Transporters

Following extracellular digestion of the meal, transporters are needed for nutrient intake as well as for maintaining pH and salt equilibrium in the gut. The worksheet “Transport” within Supporting Information S2 contains 76 coding sequences associated with this group and includes the subdivisions “amino acid and peptide transport”, “nucleotide/sugar transport”, “ABC transporters”, “permeases of the major facilitator superfamily”, “sodium solute symporters,” “lipid transporters”, “metal transporters”, “ferritins”, “aquaporins”, “monovalent cation transport and homeostasis”, “V-ATPase subunits” and “hemocyanin.”

The following highlights are indicative of the digestive tract specialization of these families. RP-23175 codes for an amino acid transporter that is significantly overexpressed in the PM, where 15 of 15 digestive reads were found. The nucleotide/sugar transporter coded by RP-2100 is overexpressed in the digestive tube, where all 757 reads were found, versus 70 in the WB. Similarly, RP-7749 is overexpressed in gut tissues. This sequence is similar to the major glucose uniporter (DpGLUT; GenBank accession number GU014570) that was functionally characterized in *D. peruvianus* [62]. A ubiquitous permease of the major facilitator superfamily (RP-28161) is overexpressed in the AM when compared to PM expression. RP-8563 is overexpressed in the digestive tissues and, principally, in the RE. This sequence is similar to that of the major midgut cation-glucose symporter (DpSGLT; GenBank accession number GU066262) functionally characterized in *D. peruvianus* [62]. Associated with water and monovalent cation transport, transcripts coding for the β-2 subunit of the Na^+^ + K^+^ ATPase were overexpressed in the AM, where all 55 reads were found. The vacuolar ATPase is important for transepithelial acidification and water transport [63]. Several of its subunits are overexpressed in the digestive tissues.

#### Protease inhibitors

Twenty-six CDS coding for protease inhibitors from the Kazal and pacifastin family are shown in this section's worksheet of Supporting Information S2. Proteins with multiple Kazal domains have been found in the AM of triatomine bugs where they act as inhibitors of blood coagulation enzymes and elastase. In some cases, their processing kinetics and crystal structure have been described [64]–[70].

The worksheet named “Prot. inhibitors” of Supporting Information S2 contains 22 CDS for proteins containing one or more Kazal domains, including previously described members of this family. RP-620, in particular, derives from an abundantly expressed transcript assembled from 4,447 digestive reads and 116 from the WB. It contains two Kazal domains and is 41% identical to the antithrombin named brasiliensin precursor of *T. brasiliensis* [71] and 39% identical to infestin 1–7 precursor [69] from *T. infestans*. RP-620 presents inhibitory activity for bovine trypsin (data not published). The transcript RP-570 contains ten Kazal-type domains seeming to play the same role as infestin 1–7 precursor in *T. infestans* [67] and brasiliensin precursor in *T. brasiliensis* [71], providing anticoagulant molecules to the *R. prolixus* digestive tract. As it contains two copies of rhodniin, a potent thrombin inhibitor [65], we cannot discard the idea that other transcripts also supply the gut with rhodniin.

Several of the Kazal members shown in Supporting Information S2 were not found transcribed in the gut tissues but provide matches to sequences previously found in sialotranscriptomes of *Rhodnius* and *Triatoma*, particularly the short single Kazal family—similar to vasotab, a potent vasodilator isolated from salivary glands of the horse fly *Hybomitra bimaculata* [72].

The pacifastin family [73], [74] is represented by four full-length and one truncated sequence, all providing matches to insect proteins annotated as pacifastin and having the Pacifastin_I PFAM domain. RP-8689 derives from an expressed transcript assembled from 75 digestive reads and 205 from the WB; it contains at least four pacifastin domains. Those pacifastin domains are not over transcribed in the gut tissues, which may suggest a physiologic role not related to digestion, possibly in the insect immune response [75].

#### Lipocalins

The lipocalin family is ubiquitous and contains a typical barrel structure, or calyx, which is often used to carry hydrophobic compounds such as lipids in an aqueous environment, thus the name lipocalin [76]. Many antihemostatic salivary proteins of triatomine bugs were found to belong to this family, including the nitric oxide (NO)-carrying heme proteins of *Rhodnius*, the biogenic amine- and adenosine-binding proteins of the same organism, and several clotting and platelet aggregation inhibitors of *Rhodnius* and *Triatoma*, which include the pallidipin and triabin proteins [76]–[83]. Contigs coding for these proteins are easily identified by the PFAM domains for nitrophorin, triabin, or lipocalin. Supporting Information S2 presents 88 CDS for this family, including an RE-specific transcript coding for RP-772, assembled from 4,242 reads from digestive tissues and only 94 from the WB. The deducted protein sequence provides many matches to salivary lipocalins of triatomines deposited in the NR database. RP-3004 matches *Galleria* gallerin, an insecticyanin homolog, and may function in lipid transport.

**Figure 7:**
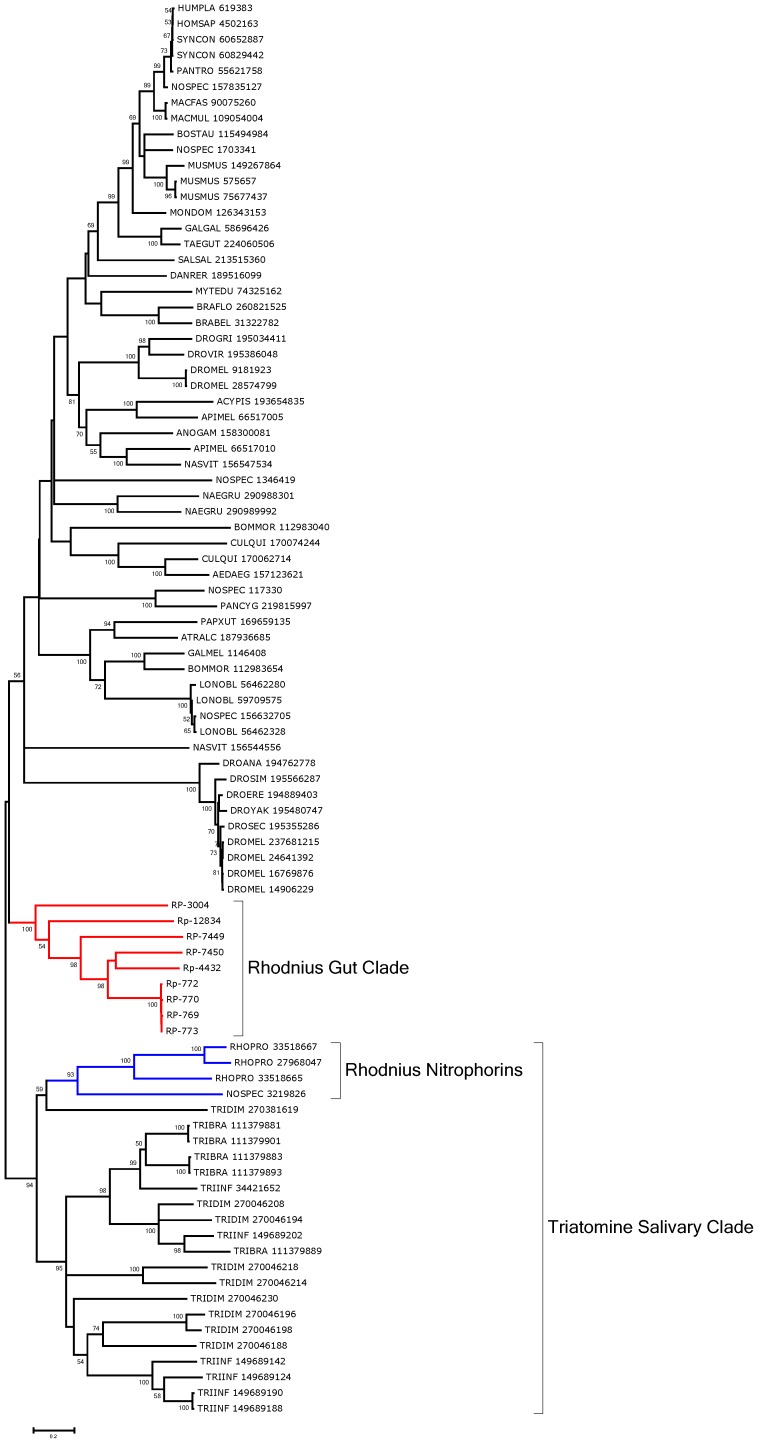
Bootstrapped phylogram of *Rhodnius prolixus* midgut lipocalins aligned with their best matches to the NR database. Bootstrap values above 50% are shown on the branches. The bottom line indicates 20% amino acid sequence divergence between the proteins. *R. prolixus* sequences are shown by the notation RP followed by a unique number. The remaining sequences, obtained from GenBank, are annotated with the first three letters of the genus name, followed by the first three letters of the species name, followed by their GenBank GI number. One thousand replicates were done for the bootstrap test using the neighbor joining test.

**Figure 8:**
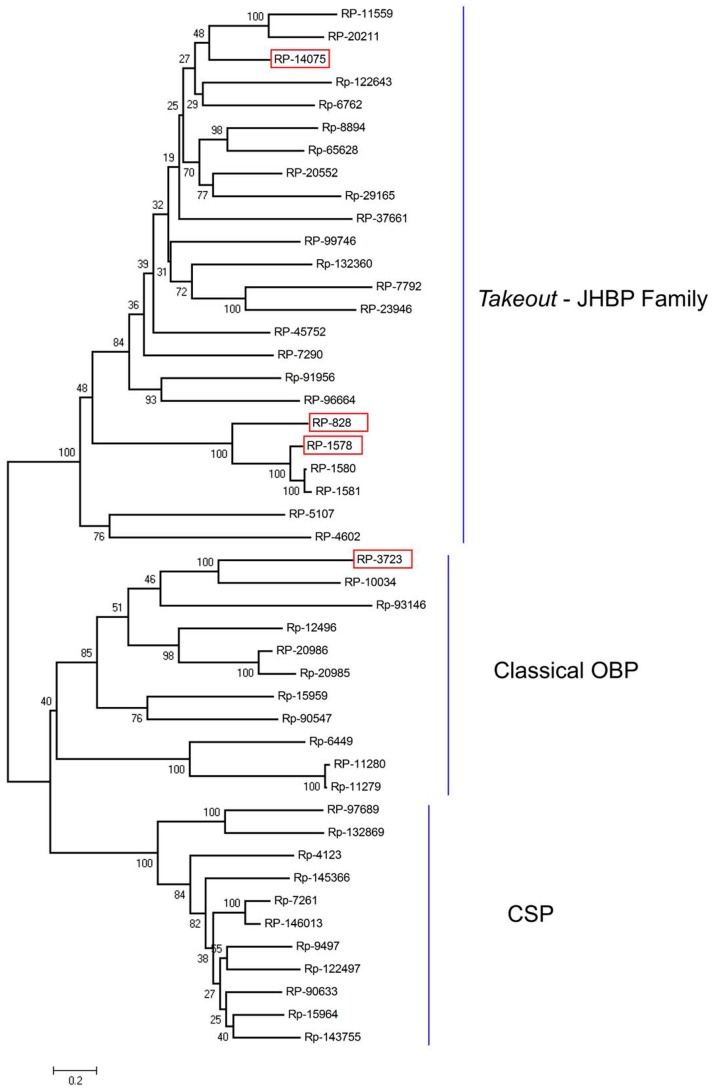
Bootstrapped phylogram of *Rhodnius prolixus* midgut *Takeout-*JHBP, Odorant Binding Protein and Chemosensorial Protein. Amino acid sequences of 46 contigs were combined to create an entry file for phylogenetic analysis in MEGA 4.0.2. An unrooted consensus neighbor joining tree was generated based on ten thousand bootstrap replicates with pairwise gap deletions using neighbor joining method. Bootstrap values lower than 50% are not shown. Red boxes indicate the over expressed proteins. JHBP: Juvenile hormone binding proteins. OBP: Odorant binding proteins. CSP: Chemosensorial proteins. For more details, see text.

Phylogenetic analysis shows a strong clade (94% support) for a common origin of the salivary lipocalins of *Rhodnius* (including nitrophorins) and the salivary lipocalins of *Triatoma* (marked Triatomine salivary clade in Fig. 7). Six lipocalins overexpressed in the gut tissues can be aligned with their best matches to the NR database and form a robust clade by themselves, indicative of gene duplication and possible gene conversion independent of the salivary clade, marked as *Rhodnius* gut clade in Fig. 8. All six transcripts have predicted signal peptides, suggesting a role in binding and transport of dietary hydrophobic compounds such as lipids from the extracellular environment.

#### Odorant-binding, takeout, juvenile hormone-binding, and chemosensorial-binding proteins

Supporting Information S2 contains CDS information for 46 contigs that contain domains from the *takeout*/juvenile hormone-binding protein (JHBP), odorant-binding protein (OBP), or chemosensorial protein (CSP) as identified by their sequence analysis and simple modular architecture research tool (SMART) or CDD matches. Four such CDS are noted in Supporting Information S2 as being overexpressed in the gut tissues, including RP-828, RP-14075, RP-3723, and RP-1578.

The phylogenetic tree for these 46 contigs showed three clearly separate groups (Fig. 8). Group I corresponds to *takeout*/JHBP (24 contigs), Group II is classical OBPs (11 contigs), and Group III is CSPs (11 contigs). The four overexpressed contigs belong either to the *takeout*/JHBP group (RP-14075, RP-1578, RP-828) or to the classical OBPs clade (RP-3723). RP-14075 and RP-7792 are members of the *takeout*/JHBP family with the two motifs characteristic of this protein family [84]. RP-828 did not show the motif 2 that characterizes a *takeout* protein and was grouped in the JHBP family. *takeout*/JHBP family proteins are carrier proteins of hydrophobic ligands and may have a role in binding or transport of signaling molecules or nutrients. JH synthesis is tightly coordinated with ingestion of a blood meal in hematophagous insects and was shown to control transcription in the midgut of *Ae. aegypti* of both trypsin [85] and chymotrypsin [86]. Although RP-3723 has been grouped in classical OBPs—which are characterized by the presence of six conserved cysteines—this transcript has only four cysteines, suggesting this is a member of CSP. In spite of its name, members of the OBP family have been ascribed roles that are not related to odor recognition, such as binding of heme by the *Rhodnius* heme-binding protein or the participation of a CSP in regeneration of *Periplaneta* legs [87]. The presence of this class of proteins overexpressed in the midgut of *R. prolixus* could suggest a role in the transport of nutrients or other molecules involved in the coordinating of physiological gut function.

#### Immunity related

Although lacking a classical adaptive immune response, insects have powerful innate immunity against several pathogens that have a cellular component involving hemocytes (leading to phagocytosis and encapsulation of pathogens), as well as a humoral response carried out by several tissues such as the fat body, midgut, trachea, and salivary glands. Humoral immunity is based on production of antimicrobial peptides (AMPs), of reactive oxygen and nitrogen species, and melanization. In this way, synthesis and secretion of antimicrobial peptides and agents to the hemolymph is generally referred to as “systemic immunity,” while the same action at the level of the barrier epithelia (as observed in the gut, for example) is generally referred to as “epithelial immunity” [88].

Production of AMPs is regulated by three primary signaling pathways, namely, Toll, IMD, and Jak/STAT [89]. In *Drosophila*, Toll responds to gram-positive bacteria and fungi, while IMD response is elicited mainly by gram-negative bacteria. This separation does not seem to be so clear in mosquitoes, where both pathways seem highly interconnected and overlapping [90]. Activation of the Toll and IMD pathways occurs upon recognition of pathogen-associated molecular patterns (PAMPs), triggering a cascade that culminates with translocation of a NF-κB-like molecule to the nucleus and hence to the production of effector molecules. It is important to note that—although in several other immune tissues, such as the fat-body, both pathways can be potentially activated— in the presence of corresponding PAMP, it is believed that in epithelial gut and tracheal responses only IMD may be activated as a consequence of proliferation of gut commensal bacteria or the presence of pathogens [88], [90], [91].

Several immune-related transcripts were identified, ranging from PAMP recognition molecules to signal transducers and effector proteins, as described below.

PAMP recognition molecules: Carbohydrate binding proteins, or lectins, could work as pathogen-recognition molecules that trigger insect defense responses [92], [93] and/or could have a role in insect feeding [94]. Several β-galactoside-binding lectins (galectins) were overexpressed in *Rhodnius* digestive tissues. RP-2747 has the Gal_lectin PFAM domain and was assembled from 428 gut-derived reads and 47 from the WB. Also, RP-15084 derives from a different gene and is overexpressed in the gut tissues.

**Figure 9:**
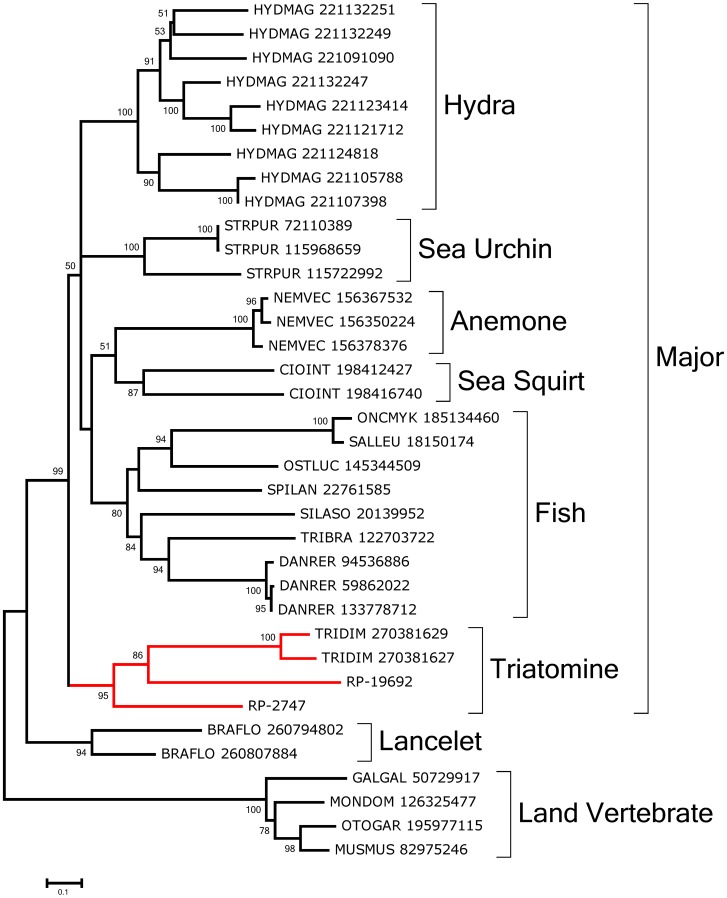
Bootstrapped phylogram of *Rhodnius prolixus* midgut lectins aligned with their best matches to the NR database. Bootstrap values above 50% are shown on the branches. The bottom line indicates 10% amino acid sequence divergence between the proteins. *R. prolixus* sequences are shown by the notation RP followed by a unique number. The remaining sequences were obtained from GenBank and are annotated with the first three letters of the genus name, followed by the first three letters of the species name, followed by their GenBank GI number. One thousand replicates were done for the bootstrap test using the neighbor joining test.

RP-2747 and RP-19692 are near full length in size and contain the CDD domain Gal_lectin. Alignment of these two sequences with their NR database matches produces the phylogram presented in Fig. 9 showing that a triatomine clade is formed with strong bootstrap support as part of a major clade formed with 99% support containing fish- and invertebrate-derived sequences. Lancelet and land vertebrate sequences lie on their own clades. The two *Triatoma dimidiata* sequences that group with the *Rhodnius* sequences have been described in a sialotranscriptome [95].

Sugar-inhibitable hemaglutinins have been described in the gut of triatomines [96], [97]. Galectins are overexpressed in gut and salivary glands of *Anopheles* infected with bacteria or *Plasmodium* [98]–[100]. It is speculated that galectins are involved in insect immune response similarly to how they are in mammals—by opsonizing bacteria and other pathogens facilitating their recognition, agglutination, and/or phagocytosis for immune-competent cells. Also, a galectin (PpGalec) has been implicated in *Leishmania major* adhesion to the midgut epithelia of *Phlebotomus papatasi*. In this case, blockage of this protein with specific antibodies leads to an important decrease in vector parasite load after six days post infection [101]. It would be interesting to assess whether any of these proteins might be involved in *T. cruzi* binding to the midgut epithelium.

RP-16133 codes for a 5′ truncated transcript producing matches against the NR database to proteins annotated as hemolectin. The best match (gi|193601326) has multiple domains, including von Willebrand, coagulation factor 5/8, TIL, and the C8 domains. Hemolectin is hemocyte-specific in *Drosophila* and is involved in the fly's clotting system [102]–[104].

Three contigs containing peptidoglycan recognition protein (PGRP) domains were also identified in the digestive tissues (Asb-69756, Asb-23314, Asb-48139). Asb-69756 and Asb-23314 do not present predicted trans-membrane regions and are likely to be soluble PGRPs. Interestingly, Asb-69756 probably presents amidase activity, as all five conserved catalytic amino acid residues are present in this protein. If that is the case, Asb-69756 could be involved in destruction of bacteria-released peptidoglycan, downregulating the bug's immune response. Asb-23314, on the other hand, is unlikely to present amidase activity, because one of the five conserved catalytic residues is missing. If that is the case, Asb-23314 could be involved in detecting peptidoglycan and activating an epithelial IMD response. The last PGRP domain containing transcript, Asb-23314, also does not present amidase activity but show a predicted transmembrane domain and is homologous to the *Drosophila* PGRP-LC (NP_729468.2). This transcript might constitute an actual PGRP-LC and may represent a receptor primarily responsible for activation of the IMD pathway in *Rhodnius*.

Immune signaling pathways: Transcripts coding for members of the immune signaling pathways were not overexpressed in gut compared to WB, but several of them showed a significant number of reads, indicating that they were operating in these tissues. Despite this, these transcripts were included in our analysis, because the midgut epithelia is the area of most intense contact between microorganisms and insects and is the only part of the triatomine body in contact with *T. cruzi*. Although it is generally accepted that the Toll pathway is not active in digestive tissues [88], [105], several contigs putatively coding for proteins from this pathway were identified—namely, a Toll receptor (Asb-44175), its adaptor protein MyD88 (Asb-69782), the kinase pelle (Asb-15772) and the pelle-associated protein pellino (Asb-24337) [106]. The evolutionarily conserved intermediate in the Toll/IL-1 signal transduction pathway [107], ECSIT (Asb-9158) and a protein from the Spätzle family (RP-45859) were identified in the transcriptome. Interestingly, contigs coding for two additional putative Toll-interacting proteins (Tollips; Asb-22553 and Asb-45642), for an inhibitor of the Toll pathway transcription factor rpDorsal Cactus (Asb-31044), the Cactus-binding protein cactin (Asb-33928), and a contig containing an NF-κB-repressing factor domain (Asb-17843) were also identified. Although these contigs were not overexpressed in the gut libraries when compared to WB, this is the first time that such a high number of Toll-related proteins were found consistently in a midgut transcriptome, suggesting that, in spite of the relative low abundance, this pathway may be of physiologic significance in gut immunity in *Rhodnius*.

In contrast to this high number of Toll-related transcripts, only one contig coding for a member of the IMD pathway was identified in the digestive tissues. It coded for the IMD negative regulator Caspar (Asb-145) [108]. This contig was highly expressed in the gut (80 reads) but also in WB (92 reads). Low expression levels also were found for the STAT pathway, where a transcript coding for a STAT (Asb-17321; 4 reads only in AM and none in WB) was identified. Together, these results suggest that all three main known immune pathways are active in the *Rhodnius* midgut.

A transcript resembling eiger was identified (Asb-21490; 21 reads from gut and 31 reads from WB). Eiger, the insect homolog of mammalian TNF, has been implicated in the immune response against extracellular pathogens [109] as well as against bacterial oral infection [110]. Eiger/TNF was suggested to be part of an ancient proof-reading pathway directed to suppress tumors in epithelial tissues based on alterations of polarity that are typical of malignant cells but that can also be found in cells that are either physically damaged or exposed to pathogens [111]. As mentioned below, there are several transcripts expressed in the gut that belong to signaling pathways related to cell polarity, indicating that sensing and control of cell polarity is a priority of *Rhodnius* intestinal cells. This could provide a link between tissue morphology and innate immunity related to intestinal pathogens.

Interestingly, three contigs putatively coding for proteins with a double-strand (ds) RNA binding domain were identified in *Rhodnius* digestive tissue libraries. One of these (Asb-16245) codes for a putative R2D2 protein. R2D2 is known to associate with Dicer-2 and is essential for channeling the siRNA generated by this protein to the RISC complex [112]. Tar RNA-binding proteins (TRBPs; Asb-26443) have a dsRNA binding domain and are structural components of the RISC complex [113]. Finally, also identified was a contig coding for a protein homologous to loquacious (Asb-21490), a protein that contains two dsRNA binding domains and was originally described as part of the miRNA generating machinery in *Drosophila* [114].

Effector proteins: Large amounts of lysozyme activity were described in the anterior and PM of *R. prolixus* [20]. Several lysozyme-coding transcripts were found to be overexpressed in gut tissues. RP-3602 was assembled from 7966 digestive reads and 619 WB-derived reads, hence being 23.1-fold overexpressed in digestive tissues. This lysozyme was previously reported as upregulated in the midgut following bacterial challenge as well as ingestion of *T. cruzi* [115]. In addition, RP-6482—although somewhat more mildly overexpressed in the digestive tissues than RP-3602—was reported to be upregulated in the FB after injection of bacteria into the hemocoel [115]. RP-24996 was the only lysozyme transcript not overexpressed in the digestive tissues. All lysozyme transcripts possess catalytic aspartate and glutamate residues except for lysozyme 2 from *T. infestans* [116]. The function of this unusual lysozyme remains to be elucidated. In *T. brasiliensis*, expression of lysozyme 1 (*lys1*) is also upregulated in the AM after feeding, with a maximum five days after blood uptake, suggesting activity against developing bacteria [117]. In addition to providing protection against airborne bacteria, these lysozymes might also function in digestion of symbiotic bacteria, which develop to high densities in the AM after blood ingestion (see section “carbohydrate digestion”). However, the number of symbionts is negatively correlated to the expression level of lysozyme and defensin genes [18], [118].

Defensins are ubiquitous antimicrobial peptides found in both invertebrates and vertebrates [119]. Insect defensins are small cationic peptides with molecular weights of about 4 kDa. They possess three disulphide bridges and contain three characteristic domains: an amino terminal flexible loop, followed by an α-helix and a carboxy-terminal anti-parallel β-sheet [120], [121]. Eight defensin sequences were included in Supporting Information S2. RP-12696 was 8.6-fold overexpressed in gut tissues; another three sequences are mildly overexpressed. Defensin A in *R. prolixus* has been shown to be upregulated in the intestine after immune activation by bacterial challenge of the hemocoel, and a much stronger upregulation was detected in the FB [122]. Similar to the upregulation of lysozyme, the transcript levels of the defensin 1 gene (*def1*) in *T. brasiliensis* is increased following blood ingestion in the AM [117], indicating activity against developing bacteria; however, insect defensins are not only active against bacteria but also interfere with development of eukaryotic parasites in the vector, e.g., *Plasmodium* and filarial helminths [122]–[124].

The SCP superfamily of proteins includes the plant pathogenesis-related protein, the secretory cysteine-rich proteins found in snake venoms, and allergen 5 found in vespid venoms [125]. These proteins may have diverse functions including as proteases and for defense. RP-7994 is a member of this family assembled from 215 reads, 184 of which are from digestive tissues.

#### Signaling pathways

Circuits of protein phosphorylation and dephosphorylation involve the concerted action of two enzyme families: protein kinases (PKs)—which add phosphate to the hydroxyl group of serine, threonine, or tyrosine—and protein phosphatases (PPs) that eventually remove such phosphate groups. Together with sequencing of genomes, the full gene complement of PKs and PPs—the kinome and phosphatome, respectively—allowed us to obtain a broader picture of signaling networks in eukaryotic cells from several organisms, but not for human disease vectors. The presence of some PKs and PPs and their probable functions in the gut of *Rhodnius* are discussed below. Additionally, some signal transduction pathways and transcription factors that regulate morphogenetic processes during development are discussed that may be involved in regenerative processes of gut physiology, as has been suggested for members of the BMP, FGF, and Wnt families of transcription factors [126].

MAPK signaling cascades: Protein tyrosine phosphatase (PTP) 4A3 (Asb-40892) is a member of dual-specificity phosphatases (DUSPs) that are able to dephosphorylate both phosphotyrosine and phosphothreonine residues in target proteins. Such enzymes are usually deactivators of mitogen-activated PK (MAPK) cascades. Such gene products recorded 21 reads from the digestive system (exclusively from PM) and none from the WB library. PTP 4A3 belongs to a subfamily of DUSPs also known as phosphatases of regenerating liver (PRLs), which play a major role in oncogenesis and are overexpressed in gastric and colorectal tumors and modulate kinases of the Erk branch of the MAPK cascade [127].

Ste20-like kinase is a serine/threonine PK, an upstream regulator of various MAPK cascades, and has 18 reads (Asb-39211), also exclusively from the PM. Ste20-like kinases function as MAPK4 enzymes, which activate the downstream cascade of MAPK3, MAP2K, and MAPK. These enzymes were originally discovered as mediators of pheromone signaling in yeast and are involved in ion transport, cytoskeleton organization, and response to osmotic stress. A member of this family, Ste20-related proline/alanine-rich kinase, is activated by hypertonicity, leading to activation of p38 and JNK MAPK cascades that phosphorylate ion transporters that regulate cell volume [128]. Activation of Ste20-like kinases also may occur in response to PAMPs such as LPS, peptidoglycan and flagelin. Variations of osmotic pressure and presence of bacteria or protozoan parasites are both major factors that govern the physiology of the *Rhodnius* midgut.

A transcript (Asb-18967) similar to MAPK-activated PK or MAPKAK2, also known as MK2, showed15 reads from the AM. This is a Ser/Thr PK activated by p38 MAPK and is involved in cell-shape change and cell adhesion. MK2 activation is also required for cytokine production during inflammatory responses [129]. It was recently demonstrated that MK2 activity is essential to cutaneous wound healing [130] and thus an enzyme of this group may be a modulator of tissue injury/immune response in AM epithelia.

Another transcript (Asb-29380) highly similar to PK C lambda/iota (PKCλ/ι) has 26 reads in all three gut libraries. This is a Ser/Thr kinase that belongs to the atypical group of PK C isoforms that are independent of calcium and diacylglycerol, which are modulators of other PK C isoforms. PKCλ/ι signals through the Rac1/MEK/ERK1,2 pathway that ultimately induces carcinogenesis in human intestine epithelial cells [131]. Its overexpression is now a prognostic for human gastric cancer [132]. A role of PKCλ/ι has been demonstrated in the establishment of epithelial cell polarity through binding members of the PAR family of proteins [133]. There is strong evidence that binding of PKCλ/ι to PAR3 and PAR6 modulate such events that depend on cell polarity as endocytosis, phosphoinositide signaling, microtubule and spindle orientation, and organization of actin cytoskeleton [134]. Thus, PKCλ/ι in the *R. prolixus* gut may be a modulator of intracellular arrangement of cytoskeleton and organelle distribution during digestive cell physiology and when it engages in its own division.

Lkb1/AMPK: An interface between cell morphology and blood digestion: The digestive system shows 86 reads of stk11 or LKB1 (Asb-6501) compared to only 27 from WB. Although Lkb1 is a PK that acts as a regulator of cell polarity and tumor suppression, it is well known as a target of cell growth regulator AMP-activated PK (AMPk; Asb-15260), a major modulator of cell energy homeostasis [135] that shows 19 reads distributed among all three gut libraries. AMPK is activated by cell signals that decrease cellular ATP. AMPK activation leads to the downregulation of ATP-consuming pathways. The major AMPK targets are glycogen synthase, acetyl-CoA carboxylase, non-muscle myosin light chain, and mTOR, which thus leads to the downregulation both of glycogen, lipid, protein synthesis and of cell polarity [136]. Studies on the Peutz-Jeghers syndrome, an autosomal gastrointestinal polyposis disorder caused by germline mutation in the *LKB1* gene, have revealed a conserved link between energy metabolism and cell polarity-dependent cell functions such as organization of the actin cytoskeleton and sorting of apical and basolateral membrane proteins to facilitate directed endosomal transportation. One molecular mechanism that links LKB1 and control of cell shape is its ability to phosphorylate the regulatory light chain of nonmuscle myosin II (MLRC), which thus regulates cytokinesis and—through myosin II—adjusts the formation of tight and adherens junctions [137]. AMPK-null mutants of *Drosophila* present several abnormalities in mitosis and cell polarity [138]. In addition, AMPK activation by energy deprivation leads to large changes in cell shape. Significant expression of LBK1 and AMPK in the gut suggests that this pathway may participate in regulation of cell polarity and energy metabolism of intestinal cells.

Another AMPK target in *Rhodnius* gut transcriptome, TOR (target of rapamycin; Asb-43781 and Asb-70063) is a PK that regulates several cellular process such as cell growth, proliferation, and survival [139]. In mosquitoes, it was shown that amino acid ingestion induces early trypsin protein synthesis coincident with activation of the TOR pathway [140], which also was implicated in control of expression of vitellogenin gene that takes place after a blood meal [141]. Although showing a low number of reads (3), these TOR transcripts suggest the presence of this nutrient and energy-sensing signaling pathway that connects the ingested meal with blood digestion and yolk protein synthesis, two different biologic processes that are separated in a time frame. Such a hypothesis must be tested at the molecular level.

Developmental regulators in adult gut. Wnt and Notch: Although the gut libraries used here were from adult females, transcripts related to two signaling pathways classically related to control of morphogenesis during development—Wnt and Notch—were identified. In adults these transcripts may be important for self-renewal or regeneration of intestinal cells [142], [143]. The transcript coding for an ortholog for *defective in proventriculus* (*dve*; Asb-11146) showed 126 reads from gut libraries (113 being from AM) and only 3 reads from WB. This gene owes its name to studies in *D. melanogaster* showing that mutants for *dve* have morphologic defects of the proventriculus [144]–[146], a region that develops at the junction of the foregut and the midgut and functions as a valve regulating the passage of food. During development, *dve* has been shown to respond to Wg (Wnt), Dpp (BMP), EGFR, and Notch signaling in the gut [144], [146], [147]; however, *dve* also has an important role in the digestive physiology of the gut. Expression of *dve* in midgut copper cells—cells that resemble absorptive mouse enterocyte cells—is necessary for acid secretion and for copper absorption [148]. Therefore, considering the high degree of sequence conservation and enrichment in adult gut tissue, *dve* is likely to perform a physiologic role in *Rhodnius*, as well. As possible regulators of *dve*, Wnt pathway elements were identified, although overexpression of Wnt pathway components in relation to the WB library was not homogeneous throughout the gut. Reads for β-catenin, a transducer of Wnt signals, were found enriched in the AM (RP-41815/Asb-1876). Furthermore, an ortholog of a RAN-binding protein, a negative regulator of the pathway (Asb-62348, with 77 reads exclusively in the RE), provides additional support for the notion that the pathway is functional. Four calmodulin transcripts were detected in the transcriptome (RP-98600, RP-96030, RP-95216, and RP-1777), but only one (RP-1777) was expressed in gut tissue. Finally, casein kinase II is likely expressed in gut tissue, as reads for the α (RP-3340) and β (RP-15495) subunits were also detected.

Interestingly, several potential Notch substrates or regulators were detected in *Rhodnius*, with a number of transcripts enriched in the gut. Notch is required for several gut-associated functions in various species. Notch regulates differentiation of endocrine cells of the mouse gut endoderm [149], regulates the switch between luminal and glandular fate in the endodermal epithelium of the chick [150], and is required in the gastrointestinal tract stem-cell niche [151]. Hairy (Asb-2287; 35 reads from gut, 25 being from AM, and 12 reads from WB) is a known element of the Notch pathway in vertebrates and invertebrates, displaying transcription repressor activity characteristic of Her proteins [152]. Another potential element of the Notch pathway is a transcript (Asb-24840, exclusively from digestive libraries; 18 reads, mainly from PM) similar to BTB/POZ domain-containing proteins bric-a-brac, Broad, and tramtrack from *D. melanogaster* that were shown to interact with the Notch path [153]. Neurofibromin (Asb-10846) is a protein shown to be regulated by Notch in the nervous system [154], [155], but it also was shown to regulate longevity and resistance to stress through cAMP regulation of mitochondrial respiration and reactive oxygen species production [156].

#### RNA-processing, translation and secretion

Posttranscriptional control of gene expression provides a prompt response to metabolic changes. In mosquitoes, translation of trypsin mRNA is regulated [157]–[159] through the TOR signaling pathway sensing the amino acid pool [140]. Other digestion-related pathways such as components of iron metabolism are also regulated at the posttranscriptional level, such as ferritin through iron regulatory proteins [160], [161]. Thus, analysis of gut-specific genes involved in translation apparatus may provide hints about posttranscriptional regulation.

There was an overall increment of expression of genes involved in RNA processing, translation, and protein secretion in the gut libraries compared to WB, probably as a consequence of the need for high rates of protein synthesis needed to cope with formation of secreted polypeptides—such as digestive enzymes and peritrophins described above—and to support epithelial cell division that must occur after a blood meal. Posttranscriptional control of gene expression provides a prompt response to metabolic changes. This has significance in comparative transcriptomics, as transcripts that do not change their abundance might still be targets of control. Regarding protein trafficking and elongation factors, some transcripts were overexpressed in all three gut libraries when compared to WB, such as the protein tyrosine phosphatase SHP1/p47 (Asb-1670), the endosomal membrane protein EMP70 (Asb-308 and Asb-663), the coatomer protein complex subunit (Asb-5008), an ADP-ribosylation factor (ARF, Asb-7450), EiF3C (Asb-610, 839, and 840), an aspartyl-tRNA synthetase (Asb-4568), and some rRNAs. Changes in ribosomal protein mRNAs have been described in the fat body of *Ae. aegypti* [162]. Similarly, we observed differential transcriptome expression in the gut. Ribosomal proteins S24 (Asb-39300), L18a (Asb-42186), L8 (Asb-18849), L21 (Asb-199), AS (Asb-1710), L19 (Asb-1947), L32 (Asb-1715), S15a (Asb-65370), P1 (Asb-5747), S16 (Asb-4689), and S29 (Asb-6829) are enriched at least five fold in the gut, while subunits S7 (Asb-1131), S25 (Asb-17734), S23 (Asb-8803), L29 (Asb-18997), and S21 (Asb-10782) are decreased. We found transcripts (Asb-1398,1400, and 1402) in the gut of *R. prolixus* with high similarities to *TRM4*, a tRNA 5-methylcytosine (m^5^C) methyltransferase with 3,163 reads from all gut libraries and 318 reads from WB. tRNA modifications have been being implicated in tRNA stability [163]–[165], translational fidelity [166]–[168], response to stress [169], [170], and control of cell growth [171]. Just recently it has been shown that one of the yeast responses to oxidative stress is the increase of m^5^C at the anticodon wobble position 34 in tRNA^Leu(CAA)^, a tRNA modification inserted by the Trm4 methyltransferase. This modification leads to selective translation of mRNA species enriched in the TTG codon, among them a specific paralog of a ribosomal protein [172]. The high expression of the TRM4-like in the gut of *R. prolixus* might be related to this fact, as its PM after a blood meal becomes a site of high oxidative stress [173], and also, as methylation of tRNAs make them more stable, it might make them available for the high turnover of protein expression following a meal. Further studies are necessary to prove these hypotheses.

In contrast, other transcripts were specifically more expressed by one of the segments of the digestive apparatus. Although most of the basal factors involved in RNA metabolism—namely splicing, polyadenylation, or translation—are expressed in all cell types, it has been shown that different isoforms can have roles in a tissue- or stage-specific manner [174], [175]. We did not observe changes in most of the translation factors, except in the RE, where there was increased expression of eukaryotic translation initiation factor 1A domain containing protein (Asb-19360; 1283 reads in Rec and 55 in WB; EF2 with 203 reads from WB and 1267 reads in Rec (Asb-1428 = RP-7150) and one isoform of initiation factor 4E (eIF4E; Asb-5727) with 13 reads from Rec and only one from WB. Two other isoforms of eIF4E are detected in the transcriptome (RP-92257 and RP-7125). RP-7125 is present in both WB and gut, while RP-92257 is only detected in WB. Despite the similarity in sequence, they correspond to different genes rather than alternative splicing, as they are encoded by different genomic contigs (Supporting Information S1). eIF4E, the cap binding protein, is a target of regulation through the TOR pathway (discussed above in the section on protein phosphorylation circuits). Interestingly, the main isoform identified in the gut is similar to the *Drosophila* eIF4E-HP, a stage-specific translational repressor [175], [176] with a conserved change of the tryptophan residue that contributes to cap recognition for a tyrosine and the lack of eIF4G/eIF-4EBP binding domain.

Regarding protein trafficking, we identify the Clathrin assembly protein AP180 (Asb-63672; 14 reads all in RE), the exocyst complex component 8 (Asb-11867; 32 reads in AM) responsible for tethering of secretory vesicles to the plasma membrane after leaving the Golgi compartment [177]; an emp24 (Asb-41987; 30 reads in PM) which is a transmembrane protein that is involved in transport of secretory proteins from ER to Golgi [178], and a guanine nucleotide exchange factor similar to *Drosophila* schizo (Asb-4883; 50 reads from AM and 1 from WB) that are known to act on ARF GTPases, which are known to regulate endocytosis [179]. While these data are consistent with ultrastructural evidence of intense protein synthesis and exocytosis in all three segments, marked tissue-specific expression of some components suggests that vesicle trafficking and protein secretion may proceed through distinct routes and be subjected to distinct pathways in each segment.

#### Detoxification

Plants produce innumerous toxic compounds to deter phytophagous insects which react with gut detoxification enzymes such as cytochrome P450s, glutathione transferases (GSTs) and other oxidases, these enzymes also participating in insecticide resistance [180]–[182]. It is expected that a blood diet would reduce the requirements for detoxification, such as that for alkaloids. On the other hand, excess heme in the diet imposes an oxidative challenge, leading to production of toxic products of lipid peroxidation, the elimination of which is accomplished by a similar array of genes [183]. In *Ae. aegypti*, some P450 genes from CYP6 and CYP9—classically involved in xenobiotic metabolism—are also transcribed in response to oxidative stress [184]. A probable member of the subfamily CYP6 [185] encoded by RP-7174 is highly expressed in all gut tissues (1519 reads), but poorly in the WB library (6 reads). Other possible members of CYP6/CYP9 subfamilies (RP-6932, RP-6776, RP-6043, RP-1459, RP-1608, RP-5848, RP-7653, RP-4925, RP-6931, RP-1613, RP-6041, and RP-11775) are significantly more expressed in gut when compared with WB. Consistent with this plethora of cytochrome P450s is the presence of transcripts that code for a cytochrome P450 reductase (RP-3922, with 173 reads from all three gut libraries versus 80 from WB), which is responsible for providing two electrons needed for activation of the oxygen molecule by a P450 enzyme during its catalytic cycle [186]. Alternatively, the same role can be fulfilled by a cytochrome *b_5_* (RP-10436), a small membrane-bound electron carrier hemoprotein [187] that, although not differentially expressed in the gut, showed up with 83 reads mainly in AM and PM. In addition to detoxification function, several insect P450s are known to be involved in steroid and lipid metabolism [188]. Final hydroxylation steps of conversion of steroid precursors into active insect ecdysteroid, 20-hydroxyecdysone, are accomplished by cytochrome P450 enzymes encoded by genes in the Halloween family [189].

GSTs are involved in detoxification by catalyzing the conjugation of glutathione with xenobiotic and toxic endogenous compounds, including products of free radical metabolism. Among the seven GST transcripts found in gut tissues, five were significantly overexpressed in all three segments of the gut, each of these being identified as a member of a different class: Zeta (RP-4940), Delta/Epsilon (RP-10873), Sigma (RP-10298 and RP-8544), and Theta (RP-3968).

Superoxide dismutase (SOD) catalyzes the dismutation of superoxide radical to hydrogen peroxide and oxygen, lowering superoxide levels and preventing formation of other reactive oxygen species and their derivatives. Its action is complemented by H_2_O_2_-eliminating enzymes such as catalase (Asb-14022 and Asb-10100), glutathione peroxidases (Asb-2104), and peroxiredoxin (Asb-10688- and Asb-10473) and its power-reducing pair thioredoxin (RP-6757). There are two major types of SOD enzymes present in animals, Cu/Zn SOD (cytoplasmic/nuclear) and Mn SOD (mitochondrial). Analysis of the *Rhodnius* transcriptome showed the presence of five SOD transcripts (RP-11791, RP-3874, RP-28439, RP-16118 and RP-1534). RP-11791, a Cu/Zn SOD, showed slightly higher expression in the gut tissues (171 reads from all three libraries and 154 reads from WB), but RP-3874, a Mn SOD, although present in the gut did not show high message levels. In *Rhodnius*, the level of hydrogen peroxide was shown to be controlled at least in part by catalase and glutathione-dependent mechanisms [190]. Also, a glutathione peroxidase activity has been shown in *Rhodnius* [191] that could be accounted for by the transcript RP-10221, 3.5-fold overexpressed in gut tissues, that seems to code for an authentic selenium-dependent enzyme. Transcripts for the rate-limiting enzyme of glutathione synthesis pathway, glutamate-cysteine ligase, were also found in gut tissues, coding both for its catalytic subunit (Asb-10777; 12 reads in gut tissues and only one in WB) and for the regulatory subunit (RP-13180; 84 reads in gut and only 47 in WB). Glutaredoxin, a small antioxidant enzyme whose active disulfide bond is reduced directly by gluthatione, is highly expressed (Asb-10150; 305 reads in the gut versus 73 in WB). Sulfate conjugation mediated by sulfotransferases (SULTs)—a mechanism of detoxification of xenobiotics as well as endogenous compounds—leads to inactivation of substrate compounds and/or increase in their water-solubility, thereby facilitating their removal from the body. Transcripts coding for these enzymes were found expressed in the gut (RP-22910, RP-11341, RP-16870, RP-97304 and RP-25906), although none were gut enriched compared to the WB. Nitration of tyrosine, in both protein-bound and free amino acid form, can readily occur in cells under oxidative/nitrosative stress, and elevated levels of nitrotyrosine have been shown to cause DNA damage or trigger apoptosis. Sulfation of nitrotyrosine occurs in cells under oxidative/nitrative stress, and it has been demonstrated that SULTs contribute to the metabolism of nitrotyrosine [192], [193]; however, although listed in the detoxification worksheet, sulfotransferases also add sulfate to proteoglycans of the extracellular matrix and therefore may be implicated in tissue remodeling as well.

Together, these data suggest that the *Rhodnius* gut has a complex network of enzymes involved in regulation of redox balance, especially involving control of the intracellular pool of reduced thiols. In spite of not being exposed to allelochemicals in food, the triatomine gut has retained significant expression of both Phase I and Phase II detoxification pathways, and the hypothesis that this may be a mechanism to ameliorate blood-induced oxidative stress needs further investigation.

The supply of reducing equivalents in the form of nicotinamide adenine dinucleotide phosphate (NADPH) is one of the most important factors in cell protection against oxidative damage. Some dehydrogenases have been shown to play a role in redox balance [194], [195], and at least one is highly overexpressed in the gut RP-6620 (614 reads in gut and 243 in WB).

The worksheet “Detox” in Supporting Information S2 presents detailed information on other cytochromes, cytochrome P-450 reductases, glutathione transferases, sulfotransferases, superoxide dismutases, short-chain dehydrogenases, and other dehydrogenases.

#### Iron and heme metabolism

Eukaryotic cells strictly control heme homeostasis by regulating biosynthesis and degradation pathways of this iron tetrapyrrol, due to its toxicity [196]. The heme biosynthesis pathway has been previously described in *R. prolixus* [197]. In fact, transcripts coding for all the enzymes that participate in this pathway have been found in the sequenced libraries. Most of these transcripts are more expressed in the WB than in the gut libraries. The exception is 5-aminolevulinate synthase (ALA-synthase, RP-2456), responsible for the rate-limiting step of heme biosynthesis, which is significantly more expressed in the digestive tissues.

Although it is already known that part of the heme molecules, released by host blood digestion, cross the digestive systems and reach the hemolymph [198], the proteins responsible for heme transport across cellular membranes remain undescribed in insects. Interestingly, transcripts coding for a protein similar to feline leukemia virus Type C receptor (FLVCR), described as a heme exporter [199], were found in the digestive libraries (Asb-18956 and Asb-197149).

In most organisms studied, heme is degraded by heme oxygenase (HO), a microsomal enzyme that catalyzes the oxidative cleavage of the tetrapyrrol ring producing α-biliverdin (BV), carbon monoxide, and iron; however, *R. prolixus* presents a unique heme-degradation pathway wherein heme is first modified by addition of two cysteinylglycine residues before cleavage of the porphyrin ring by HO, followed by trimming of the dipeptides, producing a dicysteinyl-γ-biliverdin [200]. Digestive tissues and pericardial cells present a high content of γ-BV, suggesting high HO activity [200], [201]. In this context, two distinct heme oxygenase transcripts (Asb-16264 and Asb-16263) were identified in WB and digestive tissue libraries, mostly in AM and PM, which were assigned to the same genomic contigs, suggesting that they may be generated by alternative splicing.

After heme oxidative degradation by heme oxygenase, cells face the challenge of storage and transport of the released iron without allowing oxidative damage to cells. Transferrins are extracellular proteins that bind free iron with high affinity, transferring the metal to cells by a receptor-mediated process. At least three highly expressed transcripts of transferrin (RP-6018, Asb-8333 and Asb-16041) were identified in the sequenced libraries. RP-6018 and Asb-16041 transcripts are over-represented in the WB library, whereas a high expression of Asb-8333 is also found in the digestive tissues, especially in PM and RE. Remarkably, the transcript coding for the transferrin receptor (RP-960) is more expressed in the same digestive libraries, suggesting that these tissues have to deal not only with iron molecules coming from the lumen but also with those provided from hemolymphatic transferrins. It is worthwhile to speculate that these tissues may be responsible for driving the excess circulating iron to excretion.

Another protein that plays a key role in iron metabolism is ferritin. As in vertebrates, arthropod ferritins are heteromultimers composed of two types of subunits that, in insects, are named heavy and light chain homologs (HCH and LCH, respectively). Three different transcripts of HCH subunits (RP-1172, RP-5775 and RP-7917) and two LCH subunits (RP-8697 and RP-3378) were found in the sequenced libraries. As is well known for most insect ferritins, the majority of expressed subunits present signal peptides for secretion. The exceptions are HCH (RP-7917 and RP-105633) transcripts that present a putative mitochondrial target sequence, which were not found in digestive libraries; these, as described for mammalian and *Drosophila* mitochondrial ferritins, are highly expressed in testis [202].

While most HCH and LCH subunits are ubiquitously expressed, HCH RP-1172 and LCH RP-3378 are more abundant in digestive libraries, particularly in PM, suggesting that they may be required during digestion and iron excretion processes.

Ferritin expression is posttranscriptionally regulated by intracellular iron levels due to the presence of a stem-loop structure found in the 5′ untranslated regions of mRNA named iron-responsive element (IRE). In the absence of iron, the iron regulatory protein (IRP) binds to the IRE structure, sterically blocking ferritin mRNA translation. This phenomenon is reversed when IRP specifically associates with an iron atom. IREs are present in the secreted HCH subunits RP-1172 and 5775 but not in the LCH transcripts. In fact, among all insects studied to date, only in Lepidoptera are IREs also found in LCH mRNAs [203]. Although at low level, transcripts coding for IRP (Asb-50964) were found in WB and PM libraries. The presence of all components of the IRP-IRE system suggests that the mechanism for translational control of mRNAs by iron has been conserved in this insect. Thus, a survey of other IRE-containing transcripts—especially among proteins involved in iron and heme metabolism—deserves to be done.

#### Lipid metabolism

During the blood meal, *R. prolixus* ingests a large amount of lipids such as triacylglycerol, free fatty acids and cholesterol, and the midgut is the main site of dietary lipid absorption [204]. Plasma lipids are absorbed by the PM epithelium and used in synthesis of different lipid classes that are distributed to the tissues associated with lipophorin (Lp) particles [205], [206]. Triacylglycerol digestion takes place in the PM [21], and this gut section has high expression levels of genes coding digestive lipases (e.g., RP-2369, RP-2952, RP-21001). The free fatty acids generated during this digestive reaction are absorbed by midgut epithelial cells [21], and fatty acids need to be esterified to coenzyme A (CoA) to be used by lipid metabolism pathways. This can be made by an acyl-CoA synthetase, which shows two distinct transcripts (RP-4249 and RP-24413), both more expressed in AM and PM. Alternatively, fatty acyl-CoA may be produced from *de novo* synthesis from acetate using acetyl-CoA synthetase (RP-29987), a transcript that has 31 reads in midgut and only 2 in WB. Interestingly, the AM is the major site of acetyl-CoA synthetase gene expression (and, possibly, *de novo* fatty acid synthesis), while the PM appears to be specialized in direct absorption of fatty acid from the blood meal [21]. Acyl-CoA is used in both catabolic and anabolic pathways. The midgut transcriptome shows marked expression of genes involved in β-oxidation, as 3-hydroxyacyl-CoA dehydrogenases (Asb-3668), enoyl-CoA hydratases (e.g., Asb-3371, Asb-3615), and carnitine O-acyltransferase (Asb-7656, Asb-20469), suggesting that the *Rhodnius* midgut is using fatty acid oxidation as a major source of energy. One transcript coding for a fatty acyl-CoA elongase (Asb-44706) showed only 3 reads from WB and 123 reads from gut, mainly from Rec (119 reads), possibly related to synthesis of long-chain hydrocarbons that are components of the wax layer that covers the wall of the hindgut [207].

Expression of the sterol regulatory element-binding-protein homolog (Asb-14714; 17 reads in gut versus 4 in WB), especially in AM and PM, suggest that the *Rhodnius* midgut is able to make *de novo* lipid synthesis, as this transcription factor induces expression of acetyl-CoA carboxylase and fatty acid synthase in *Drosophila* [208]. As this transcriptome was made from organs dissected from both unfed and blood-fed insects, it is not possible to determine when fatty acid synthesis would occur. The *Rhodnius* midgut also expresses the NPC1b homolog (Asb-2638; 55 reads in gut and 19 in WB), especially in anterior and PM, organs involved in absorption of cholesterol, which is transferred to lipophorin similarly to what happens with other lipids (Entringer et al., unpublished results). NPC1b protein is essential to absorption of ingested cholesterol by midgut cells in *Drosophila* [209]. High expression of transcripts coding for hydroxysteroid 17-β dehydrogenase (Asb-5710) and C-4 sterol methyl oxidase (Asb-5381) indicate that ingested cholesterol may be further metabolized into other sterols.

The midgut transcriptome also reveals upregulation of genes involved in complex lipid metabolism, as fatty acid desaturase (Asb-1771), glycerophosphoryl diester phosphodiesterase (Asb-14330), and diacylglycerol O-acyltransferase (Asb-1487). There are also high expression levels of genes that participate specifically in phospholipid biosynthesis, such as sphingomyelin phosphodiesterase (Asb-1419 and Asb-1420, with 133 reads in all gut libraries and 60 in WB), that catalyzes the hydrolysis of sphingomyelin to ceramide, which may be further metabolized to bioactive lipids, as sphingosine and sphingosine 1-phosphate. A transcript similar to a choline kinase (Asb-6000) also showed high expression (65 reads in gut, mainly AM and PM, and 25 in WB). This enzyme phosphorylates choline to generate phosphoryl choline, which is the first step in the so-called Kennedy pathway for phosphatidylcholine synthesis [210]. High choline kinase activity has been implicated in tumor development, possibly by regulating Akt phosphorylation, thereby promoting cell survival and proliferation [211], a role that could be critical for tissues that need high cell-renewal rates, such as digestive epithelia. Phospholipid transfer proteins (PL-TPs) such as the phosphatidylinositol transfer protein (Asb-15071; Asb-40276) are expressed in the *Rhodnius* midgut. These proteins transport phospholipid inside the cells—transferring either phosphatidylinositol or phosphatidylcholine between membranes [212]—and contribute to releasing secretory granules and secreting of vesicles from the trans-Golgi network [213]. These proteins probably are related to phospholipid synthesis needed to generate membranes of secretory vesicles to be used in the formation of the perimicrovillar membranes or to be transferred to lipophorin and exported to the hemocoel [214].

Another possible function of phospholipids in the gut involves their signaling role as a source of bioactive lipid molecules through the action of phospholipases (PLs). PLs work as digestive hydrolases but also comprise a heterogeneous group of ubiquitous enzymes involved in such diverse processes as membrane homeostasis, signal transduction, and generation of bioactive molecules [215]. One product of PL action (specifically PLA2) is lysophosphatidylcholine, which is a component of saliva and feces of *R. prolixus* [216]. Only one transcript coding for a lysophospholipase like-1 is overexpressed in gut (RP-7099; 48 reads mainly from AM and PM libraries, and 9 from WB) but several other candidate PLs show significant expression levels in *Rhodnius* gut: RP-1587 (PLC C), RP- 4722 (lysophospholipase), RP-5116 (PLD), RP-6129 (PLB), and RP- 7274 (PL/carboxyhydrolase). Signaling by lysophosphatidic acid is turned off [217]–[219] by means of lysophosphatidic acid acyltransferase (LPAAT, RP-10018), showing 19 reads in gut, most in PM, and 5 reads in WB. As already mentioned, PL-TPs transport phospholipids from their site of synthesis to other cell membranes, but also have been related to phospholipase C-mediated inositol signaling, PI3 kinase-mediated phosphorylation of PIP2 to PIP3, and formation of leukotrienes and lysophospholipids [213], [220], [221]. Four transcripts coding for PL-TPs with SEC14 domain (RP-6243, RP-6447, RP-21186 and RP-12057) were overexpressed in gut tissues, highlighting the complexity of PL metabolism and trafficking in these tissues.

In different cell types, lipids are stored in cytoplasmic organelles termed lipid droplets (LDs). LDs store fatty acids and cholesterol as neutral lipids, predominantly triglycerides (TG), cholesterol esters, and diacylglycerol, surrounded by a phospholipid monolayer and coated with a complex set of proteins [222]. Perilipins (Rp-2667; 635 reads from gut and only 21 from WB, overexpressed in all gut tissues, but especially in RE) are proteins characteristic of LDs. Proteins belonging to the PAT family are now collectively referred to as perilipins, including proteins previously known as adipophilin and tail-interacting proteins [223]. Perilipins regulate lipase access to LDs according to cell metabolic needs [224], [225]. In the past few years, it became clear that LDs are not simple lipid storage depots but rather complex organelles involved in multiple cellular functions such as lipid biosynthesis and catabolism, signal transduction, and energy and cholesterol homeostasis. Proper use of both dietary lipid and lipid synthesized *de novo* from other metabolic precursors involves absorption, intracellular trafficking inside gut epithelia, and transfer to the hemocoel—a chain of events that almost certainly must involve LDs.

#### Amino acid metabolism

Proteins are largely the most abundant component of vertebrate blood, and therefore, its digestion is a formidable source of amino acids. When transcripts most abundantly expressed in the midgut were analyzed, a marked predominance of enzymes related to amino acid degradation/gluconeogenesis was found. From 28 transcripts related to amino acid metabolism that were significantly overexpressed in the gut, 21 coded for degradation pathways. The first biochemical reaction in most of amino acid degradation pathways is catalyzed by transaminases, which transfer –NH_2_ to ketoacids (mainly oxaloacetate, α-ketoglutarate rendering aspartate or glutamate, respectively, and to pyruvate rendering alanine) or dehydrogenases that transfer –NH_2_ to H_2_O rendering NH_4_^+^. Among the transaminases, it is remarkable that broad-spectrum transaminases, mainly tyrosine aminotransferase (TAT; RP-18771 slightly overexpressed in gut tissues) and aspartate aminotransferase (ASAT; Asb-40230; RP-5603) are present in all three sections of the gut, indicating the presence of a robust transamination network. Typical ASATs constitute a node linking alanine, aspartate, glutamate, cysteine, methionine, arginine, proline, tyrosine, phenylalanine and eventually tryptophan metabolic pathways, while typical TATs are restricted to cysteine, methionine, tyrosine, and phenylalanine. Possible participation of TATs in the metabolism of alanine, aspartate and glutamate cannot be ruled out, however, because this involvement was also described in some cases. The presence of a branched chain amino acid aminotransferase (which transfers –NH_2_ from isoleucine, leucine and valine to α-ketoglutarate, Asb-5595) also contributes to connect virtually all amino acid metabolic pathways. The transamination network seems to be reinforced by an aromatic amino acid aminotransferase (AAAT; RP-6050) which connects the tyrosine, phenylalanine, cysteine, and methionine metabolic pathways. The presence of mRNA for phosphoserine aminotransferase (Asb-13727, Asb-13728, Asb-13729), a more specific enzyme participating in the glycine, serine and threonine metabolism, was also detected with higher expression levels in all three gut segments. Interestingly, ASAT is more expressed in the AM and RE, while the TAT and AAAT seem to be more expressed in the PM. As mentioned above, different transamination proFile S are able to interlink different amino acid metabolic pathways. Changes in the transaminase profile can determine changes in the “channeling” of substrates to different metabolic pathways. Additionally, contigs coding for proteins with similarity to glutamate/leucine/phenylalanine/valine dehydrogenases were consistently expressed (Asb-7486 and Asb-7477). As a whole, the transamination/deamination network is also responsible for linking most amino acid degradation pathways with the tricarboxylic acid cycle (as mentioned above, intermediates such as oxaloacetate and α-ketoglutarate are main –NH_2_ acceptors) and with glycolysis (being pyruvate, a main glycolytic intermediate, another main –NH_2_ acceptor).

Enzymes related to the pathway for degradation of aromatic amino acids were over-represented (8 contigs) with very large numbers of reads in all three midgut libraries. The presence of roughly homogeneous quantities of mRNAs coding for a phenylalanine hydroxylase (Asb-19784, Asb-19783), 4-hydroxyphenylpyruvate dioxygenase (Asb-5323, Asb-5324), homogentisate 1,2-dioxygenase (Asb-3986, Asb-3918), maleylacetoacetate isomerase (Asb-2192), and fumarylacetoacetase (Asb-3548) are observed in the three sections of the midgut. This result suggests that tyrosine is degraded to acetoacetate (an intermediate common to the lipid degradation pathway, which is why this amino acid is ketogenic) and fumarate (an intermediate of Citric of acid cycle) all along the digestive tube. The presence of an aromatic amino acid decarboxylase, on the other hand, although only 1.6 times overexpressed, could account for an alternative fate for these amino acids, channeling then into the melanization pathway. This hypothesis is reinforced by the overexpression of a transcript similar to tan (RP-5882; 134 reads from digestive libraries and only 24 reads from WB), an enzyme that in *Drosophila* was shown to catalyze the hydrolysis of N-β-alanyl dopamine (NBAD) to dopamine during cuticle melanization [226].

Although tryptophan is an essential amino acid and less abundant in the composition of most proteins, its degradation pathway is marked over-represented, with 5 contigs coding for enzymes overexpressed in gut libraries (kynurenine formamidase, Asb-1659, Asb-1660; kynurenine 3-monooxygenase, Asb-670; kynurenine-oxoglutarate transaminase, Asb-9304, Asb-9305). The exception is tryptophan dioxygenase (RP-58688; 51 reads from WB and 2 from gut tissues), the first enzyme of the pathway, which is generally considered to be rate limiting. This could reflect that expression of this transcript occurs over a short period of time at very specific moments and that the time points used to isolate mRNA for the libraries lost this point. Alternatively, one should think that an alternative oxygenase could be involved in the formation of the second intermediate in the path, n-formyl-kynurenine, substrate of kynurenine formamidase (657 reads in the RE and 702 reads from WB). The tryptophan degradation pathway has been ascribed to an immunosuppressive role, acting through limiting lymphocyte proliferation by reducing availability of this essential amino acid [227]. In addition, xanthurenic acid—an intermediate in this pathway linked to ommochrome formation—induces gametogenesis of *Plasmodium* in the gut of mosquitoes [228]. Recently, xanthurenic acid was shown to act as an antioxidant, protecting midgut epithelia against heme-induced damage [229]. It was also shown that blocking tryptophan degradation impaired resistance of mammalian cells against infection by *T. cruzi*, which were shown to be sensitive to intermediates in the pathway, namely hydroxykynurenine [230].

In contrast, proline and serine biosynthesis seems to be upregulated in the midgut. Delta-1-pyrroline-5-carboxylate synthetase (Asb-13754, 43 reads from PM libraries and 34 reads from WB) is a bifunctional enzyme that catalyzes the two initial steps of proline biosynthesis from glutamate (the conversion of glutamate into glutamyl phosphate and its further conversion into glutamate-5-semialdehyde) and is usually considered to limit the flux in the pathway [231]. Glutamate-5-semialdehyde is interconverted spontaneously into Delta-1-pyrroline-5-carboxylate, which is the substrate of the Delta-1-pyrroline-5-carboxylate reductase (Asb-23468 and Asb-17599), which catalyzes the synthesis of proline. mRNAs for this enzyme were also found in the midgut, showing that the full proline biosynthetic pathway seems to be functional. Conversely, Delta-1-pyrroline-5-carboxylate dehydrogenase (Asb-45634), an enzyme from the proline degradation pathway, presents only 2 reads in gut and 22 from WB. Together, these data led us to speculate that carbon skeletons of amino acids may be exported as proline from the midgut, as this imino acid is known to be widely used as an energy substrate by insect tissues, including in flight muscle [232]. Interestingly, no genes coding for enzymes connecting the arginine and proline metabolism or related to biosynthesis or degradation of arginine (included those corresponding to the urea cycle) seem to be expressed.

Finally, two speculations arose from the data obtained on amino acid metabolism. The first relates to the presence of mRNAs coding for histidine decarboxylases (Asb-2365). Histidine decarboxylase converts histidine into histamine, which is an intermediate of a metabolic pathway connecting histidine to aspartate and glutamate metabolism; however, no other genes coding enzymes for this pathway were evidenced. Besides its very well-known involvement in intercellular communication, acting as a mediator of allergic responses in mammals, histamine is a modulator of digestive processes, being an activator factor of the secretion of HCl and pepsinogen in mammals. It is also intriguing that histamine is necessary for vision and mechanoreceptor functions in insects, and this excess metabolic histamine may provide a reserve for these needs [233].

Second, the presence of a possible glutamate decarboxylase (Asb-12477) and a 4-aminobutyrate aminotransferase (Asb-11677) in the midgut strongly suggest the conversion of glutamate into γ-amino butyric acid and its further conversion into succinate semialdehyde. To be fully oxidized, succinate semialdehyde should be converted into the Krebs cycle intermediate succinate by a succinate semialdehyde dehydrogenase. No mRNAs coding for this enzyme were detected. As hypothesized for other metabolic routes, it could be the case that γ-amino butyric acid and/or succinate semialdehyde are transported to target cells that are able to metabolize them. The possibility that these metabolites could be acting as messengers for intercellular communication should be also considered.

### Viruses, *Wolbachia*, and transposable elements

The polyprotein for a picornavirus similar to the honey bee slow paralysis virus is found expressed in the WB, AM, and RE (Asb-4202). This viral sequence was not found in the genome scaffolds, suggesting it may not be part of the insect genome. The DNA helicase of a virus similar to *Cotesia vestalis bracovirus* was also found in (Asb-64576); other transcripts matching *Cotesia* virus were also found. Several phage proteins were also identified, and these could derive from bacterial transcripts. For example, Asb-15041 is 70% identical to a phage from a *Wolbachia* endosymbiont, but this is mapped to *R. prolixus* genome in contig 5802 and could represent a horizontal transfer. Also, 80 transcripts best-matched bacterial proteins (presented in worksheet “Bacteria Virus TE” in Supporting Information S1), many of which appear to be mapped to the genomic contig 17820 (assembly version 3.0) including several sequences best matching *Wolbachia* endosymbionts. These could be interpreted as contaminant microorganisms present in both the colonies used to make the transcriptome reported here and the colonies that were used to sequence the genome. As these colonies have been kept in captivity for decades and were obtained independently from very distant places, this would make this *Wolbachia* a strong symbiont candidate. If these genomic contigs do not represent artifacts of genome assembly, this could represent an insertion of *Wolbachia* genetic material common to both *Rhodnius* strains, as has been reported for several insect species, where segments as large as the entire genome of a bacteria are found inserted into the genome of the arthropod [234].

Abundant transcripts coding for TEs, on the other hand, are found incorporated into the genome, as expected. In particular class I TE sequences of the families Gypsy, Bell, Line, and Copia are abundant. The class II (cut and paste) transposons are also particularly abundant, with expressed sequence tags coding for full-length transposases of a Mariner element (Asb-69103, RP-85192), suggesting active transposition. PIF/Harbinger elements are also transcribed (Asb-6109).

### Oddities

One-zinc-dependent metalloprotease was detected (RP-9242), which may be involved in cleaving growth factors or extracellular matrix components.

Transcript Asb-10133 codes for a small protein of 80 amino acids, highly expressed in the Rec and homologous to the bladder cancer-associated protein (BLCAP/Bc10) downregulated during invasive cancer growth in bladder [235]. The function of this protein is unknown, but its expression is characteristic of stratified epithelia, also found in *Rhodnius* hindgut.

Transcript Asb-820 codes for a pantheteinase overexpressed in all three segments (1025 reads from gut libraries and 197 from WB). Enzymes belonging to this gene family are involved in vitamin recycling, both hydrolyzing biotinyl-peptides, generating free biotin, and transferring biotin to acceptor proteins. These proteins could in this way make biotin from the diet available to allow the insect to synthesize its own biotin-dependent enzymes, such as carboxylases.

### Conclusions

Currently, the *R. prolixus* genome has been sequenced with a 9× coverage. Transcripts reported here helped to obtain the predicted gene set that is available at vector base homepage (https://www.vectorbase.org/organisms/rhodnius-prolixus) and were also used to support the manual annotation effort. The transcriptome described here represents a significant increase in the amount of information on *Rhodnius* genome, with 2,475 near full-length coding sequences being deposited to GenBank. Several transcripts corresponding to functions that were expected— such as digestive enzymes and transporters—appeared in large numbers, and some findings have added new data that can help to understand aspects of the digestive physiology of this insect and its interaction with intestinal microbiota and trypanosomatids, as well as generate new working hypotheses for future research. The differential expression data here reported is based in a single sample comparison and further results using microarray or RNAseq data are required for their validation.

## Supporting Information

Figure S1 Protein extracts fractionated on a 4–12% NuPAGE gels, revealed by SafeStain Coomassie Blue.
(DOCX)

Supporting Information S1 Hyperlinked spreadsheet with contig assemblies.
(DOCX)

Supporting Information S2 Hyperlinked spreadsheet with deducted coding sequences.
(XLS)

Supporting Information S3 Hyperlinked spreadsheet with deducted coding sequences and details of the proteomic match.
(XLSX)

Table S1 Table exhibiting functional class distribution of the proteins confirmed by proteomic approach.
(DOCX)
