# Supplementary material for: An Insight into the Transcriptome of the Digestive Tract of the Bloodsucking Bug, Rhodnius prolixus
Source: PLoS Negl Trop Dis. 2014 Jan 9;8(1):e2594. doi: 10.1371/journal.pntd.0002594 (PMC3886914; doi:10.1371/journal.pntd.0002594)
Supplement: Figure S1 — Protein extracts fractionated on a 4–12% NuPAGE gels, revealed by SafeStain Coomassie Blue. (DOCX) [file pntd.0002594.s001.docx]

**Supplemental Figure S1**

Protein extracts fractionated on a 4-12% NuPAGE gels, revealed by SafeStain Coomassie Blue. Band numbering identifies the source of ions submitted to mass spectrometric analysis confirming the existence of proteins listed in Supplemental Files 2 and 3. AM, anterior midgut; PM, posterior midgut and RE, rectum.

Soluble proteins

Membrane proteins


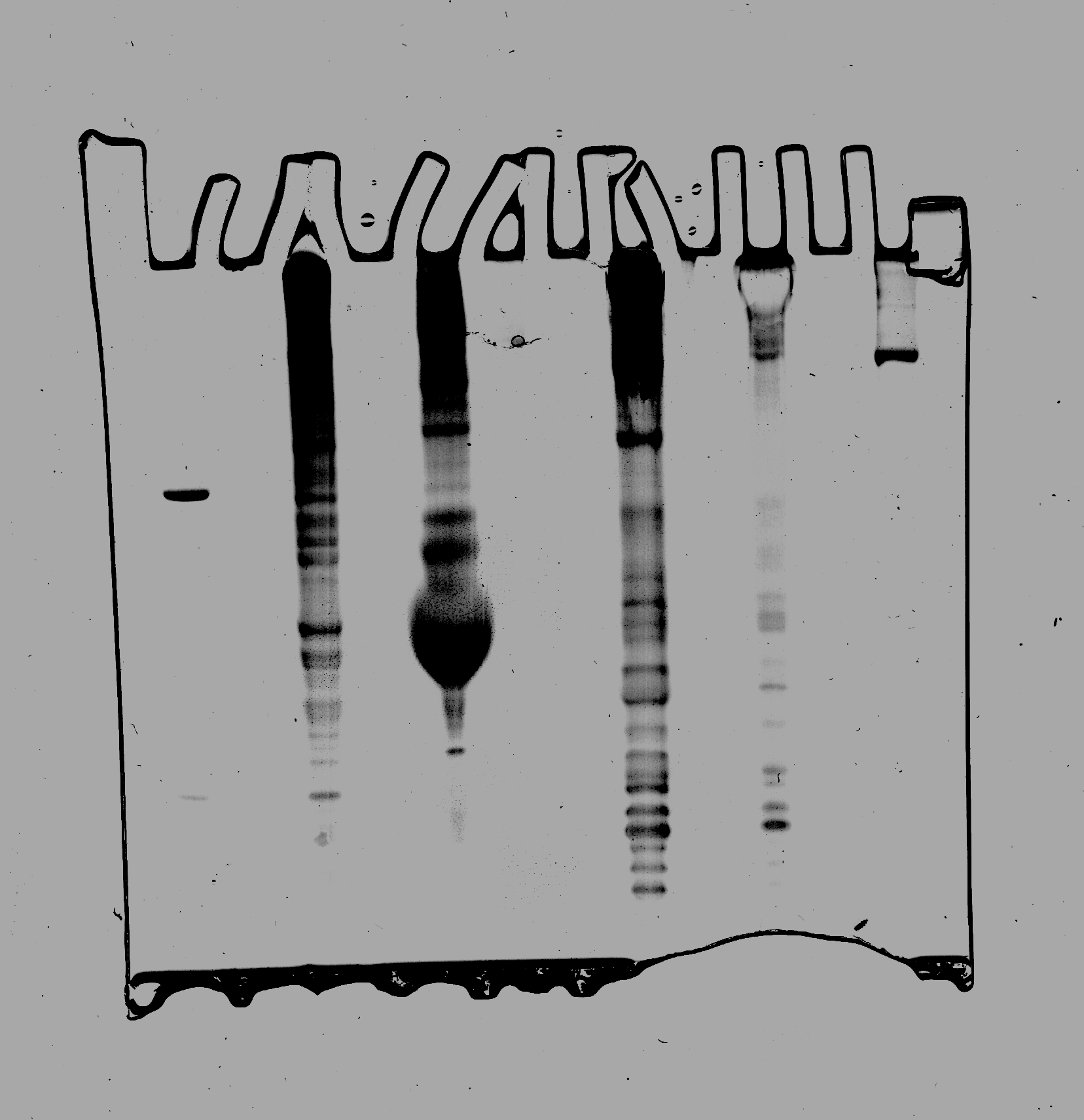

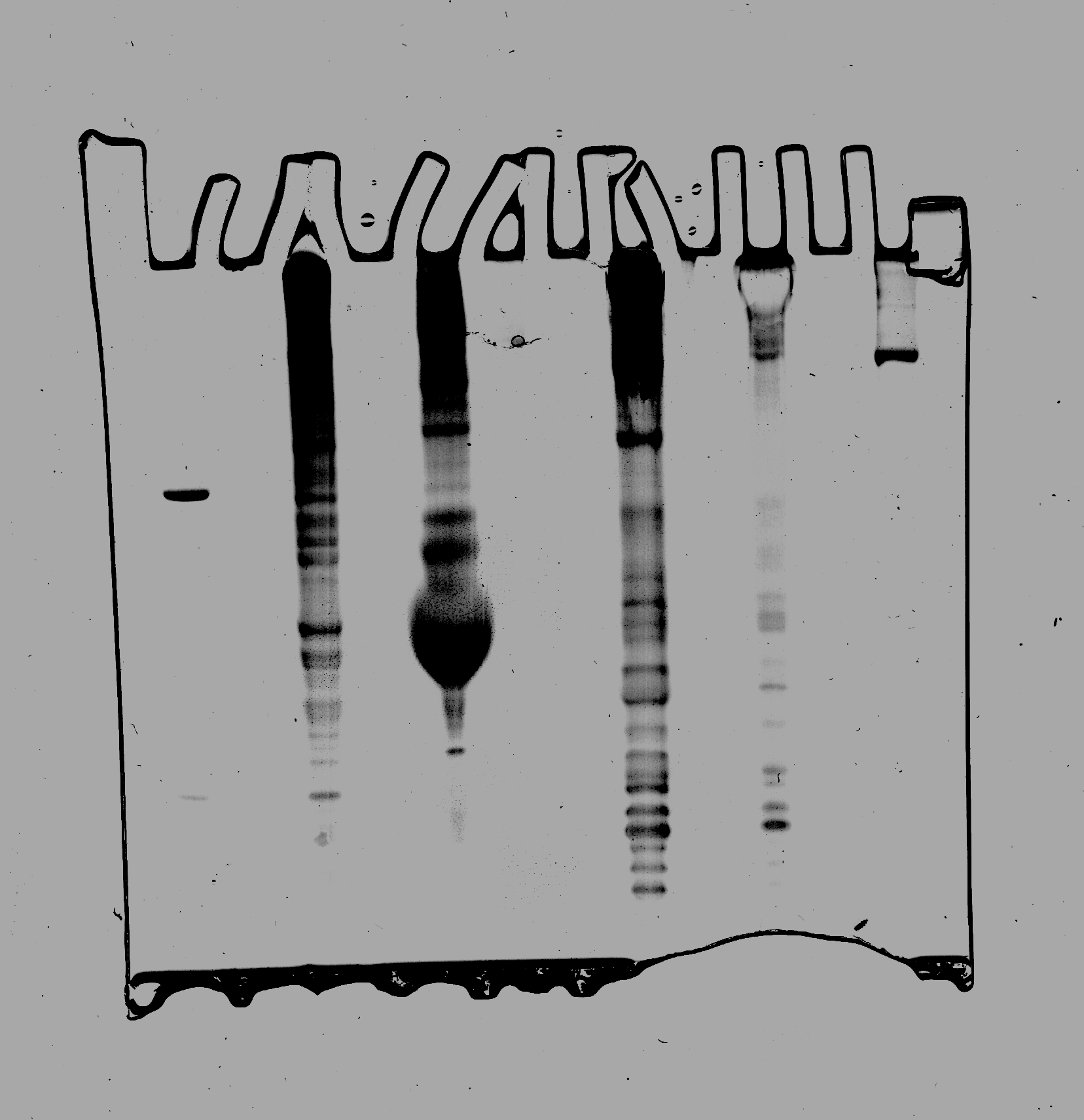


**AM**

**PM**

**RE**


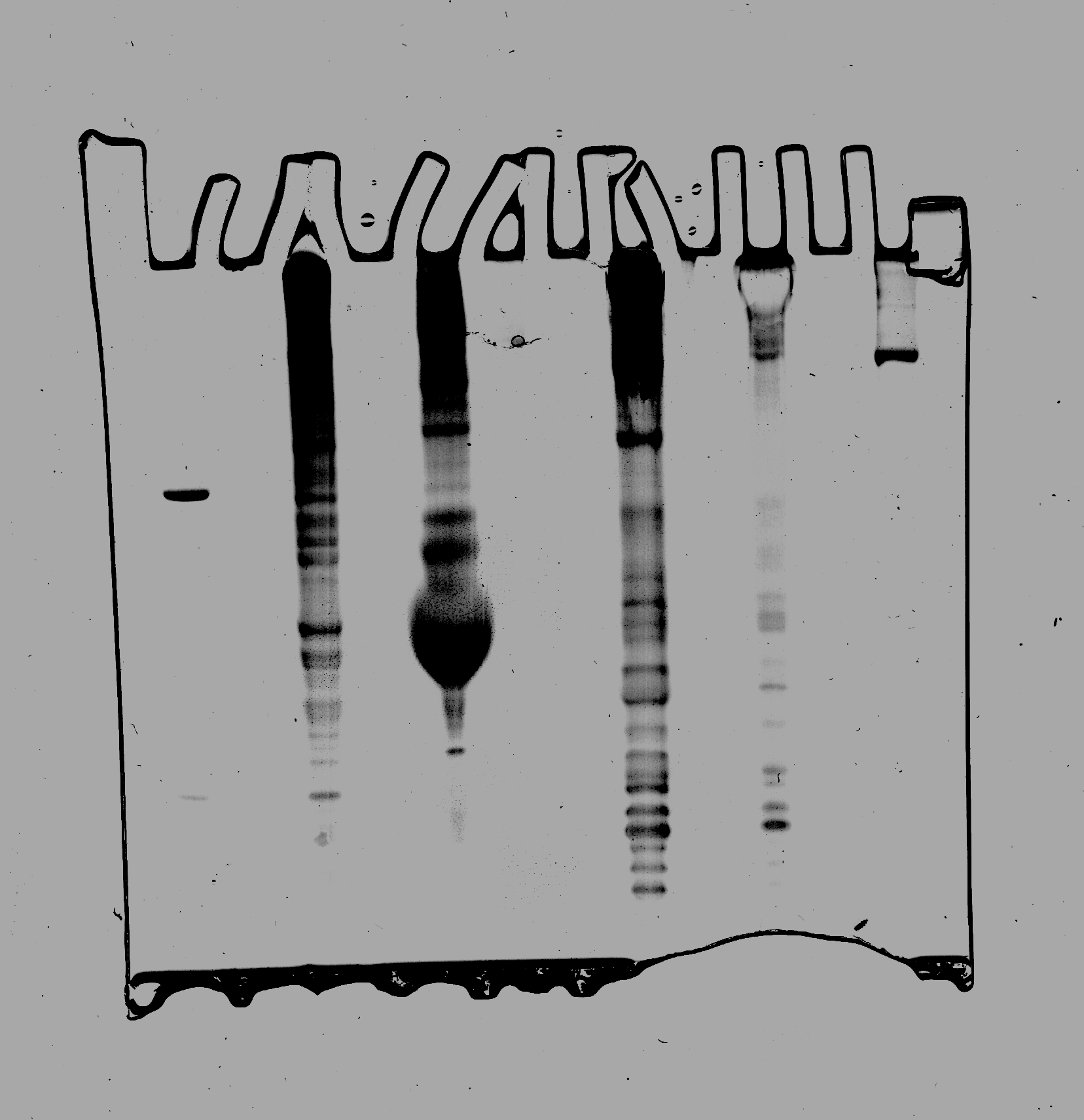


02

01

03

04

16

24

08

23

25

10

09

11

12

13

14

15

07

06

05

17

18

22

21

19

20

116

97

66

55

kDa

200

36


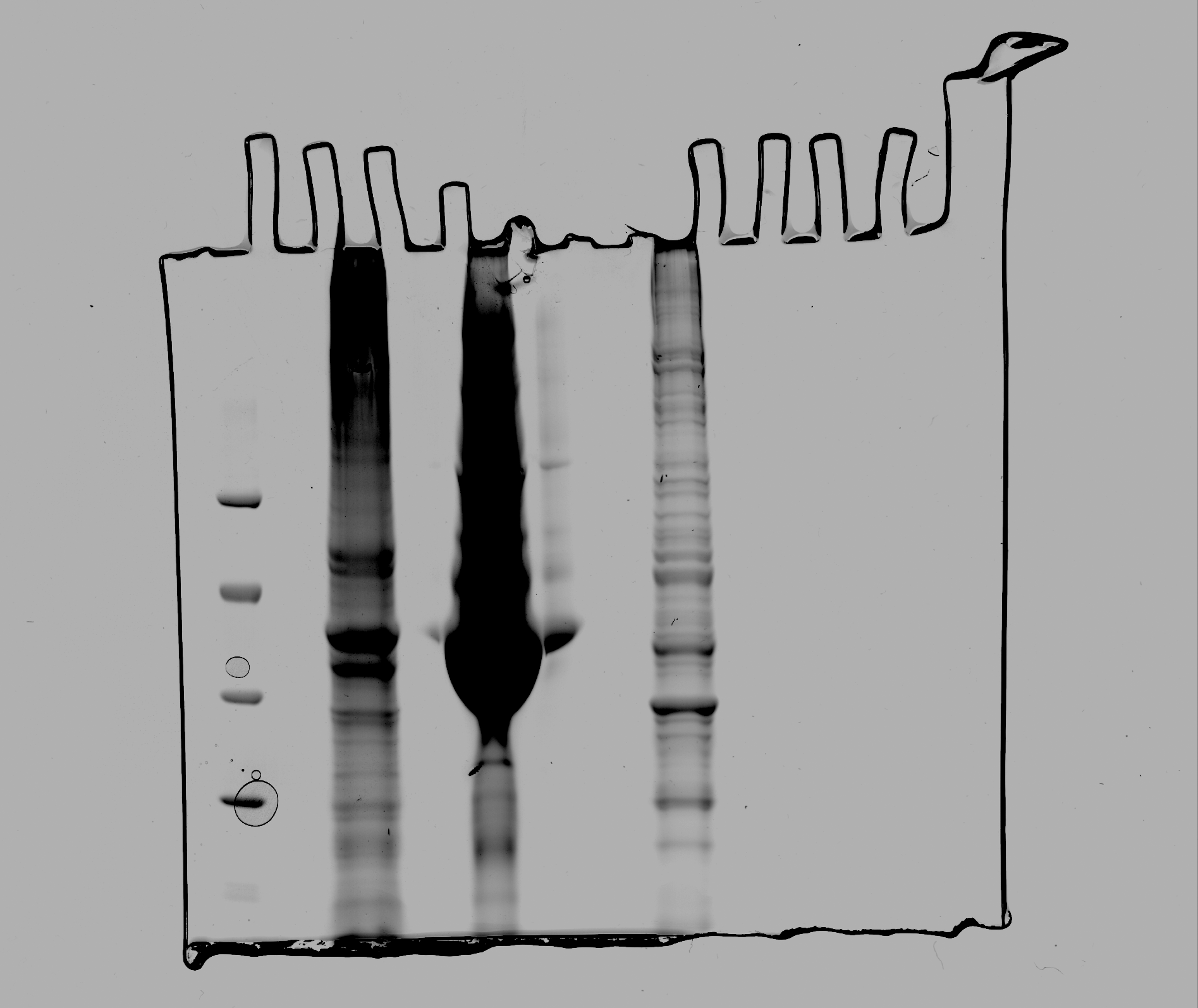

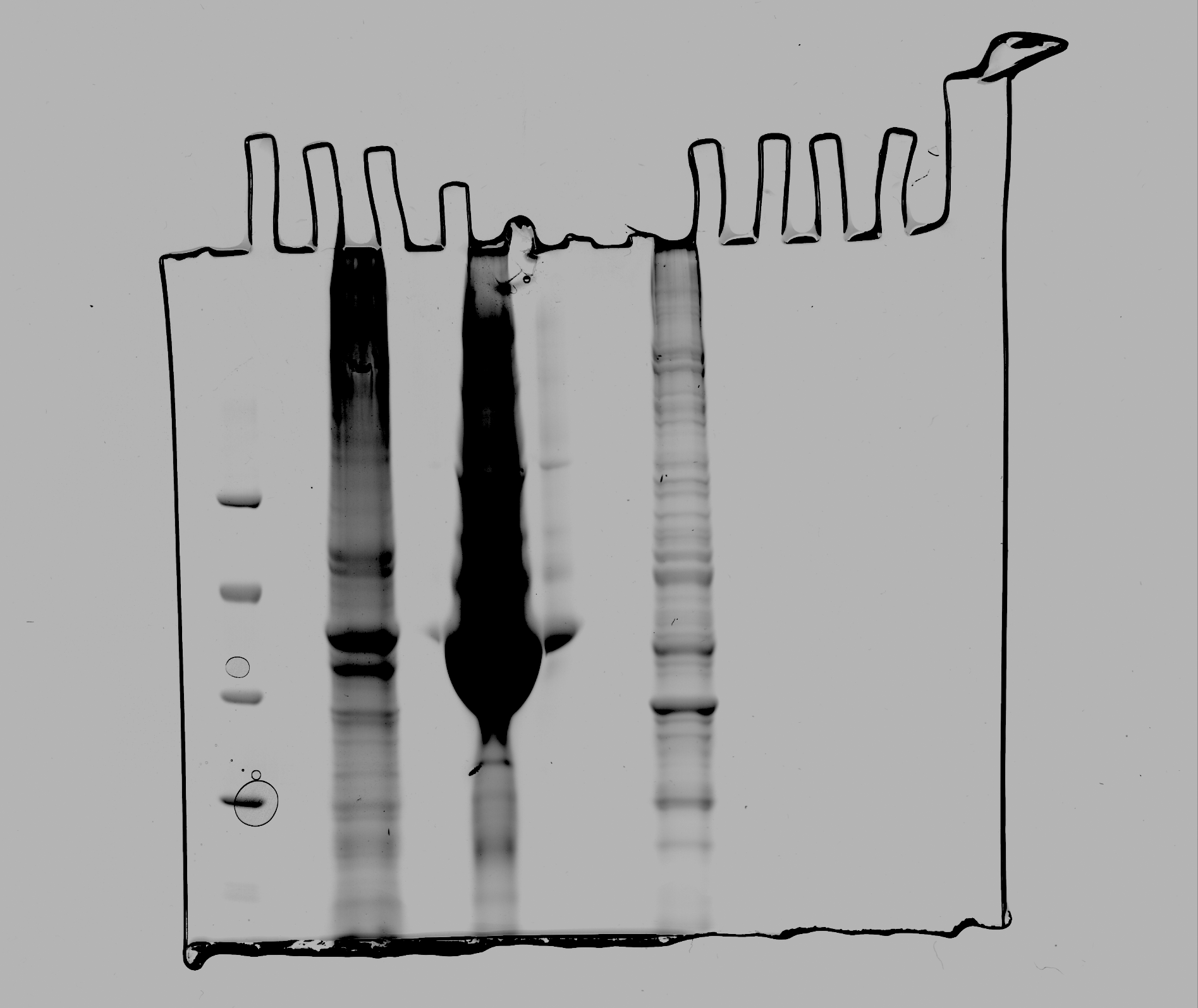

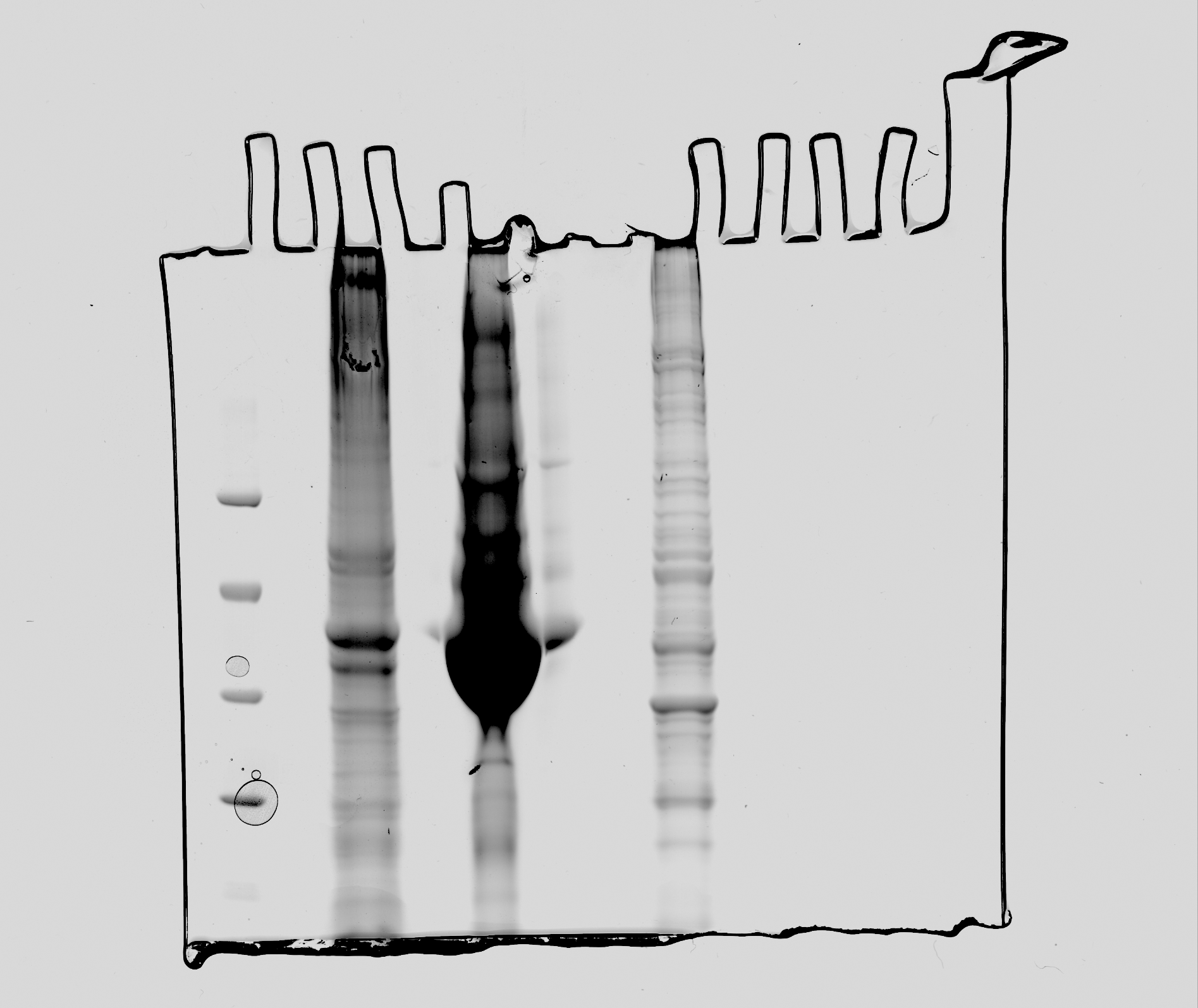


02

01

03

04

08

23

07

06

05

22

21

09

10

11

12

13

14

15

16

17

18

19

24

25 A

26

27

28A

29

30

31

32

33

34

35

36

37

38

39

40

41

42

43

44

45

46

47

48

49

50

51

52

53

54

55

56

57

58

59

60

25 B

28B

**AM**

**PM**

**RE**

200

116

97

66

55

36

kDa

20
