# Supplementary material for: An Insight into the Transcriptome of the Digestive Tract of the Bloodsucking Bug, Rhodnius prolixus
Source: PLoS Negl Trop Dis. 2014 Jan 9;8(1):e2594. doi: 10.1371/journal.pntd.0002594 (PMC3886914; doi:10.1371/journal.pntd.0002594)
Supplement: Supporting Information S1 — Hyperlinked spreadsheet with contig assemblies. (DOCX) [file pntd.0002594.s002.docx]

Supplemental File S1 – Hyperlinked Spreadsheet with Contig Assemblies

http://exon.niaid.nih.gov/transcriptome/R_prolixus_454/S1/Rp-S1-web.xlsx
