# Supplementary material for: An Insight into the Transcriptome of the Digestive Tract of the Bloodsucking Bug, Rhodnius prolixus
Source: PLoS Negl Trop Dis. 2014 Jan 9;8(1):e2594. doi: 10.1371/journal.pntd.0002594 (PMC3886914; doi:10.1371/journal.pntd.0002594)
Supplement: Table S1 — Table exhibiting functional class distribution of the proteins confirmed by proteomic approach. (DOCX) [file pntd.0002594.s005.docx]

**Supplemental Table S1:**  Functional classification of deduced proteins of *Rhodnius prolixus* that have their existence confirmed by proteomic approach.

| **Class** |  |
| --- | --- |
| **Associated with digestive physiology** | |
| Oxidant metabolism/detoxification | 8 |
| Other secreted | 1 |
| Digestive enzymes | 14 |
| Odorant binding proteins | 1 |
| Peritrophins | 3 |
| **Associated with cellular function** | |
| Cytoskeletal | 28 |
| Transcription machinery | 3 |
| ***Unknown, conserved*** | **8** |
| Metabolism, amino acid | 5 |
| Extracellular matrix/cell adhesion | 5 |
| Metabolism, lipid | 12 |
| Metabolism, energy | 22 |
| Protein modification machinery | 25 |
| Signal transduction | 10 |
| Nuclear regulation | 5 |
| Transcription factor | 11 |
| Protein export machinery | 42 |
| Metabolism, carbohydrate | 12 |
| Metabolism, nucleic acid | 3 |
| Metabolism, intermediate | 5 |
| Proteasome machinery | 2 |
| Secreted immune | 1 |
| Total | 226 |
